# SEMA3B‐AS1 suppresses colorectal carcinoma progression by inhibiting Semaphorin 3B‐dependent VEGF signaling pathway activation

**DOI:** 10.1002/mco2.365

**Published:** 2023-09-10

**Authors:** Yi‐Qing Wang, Hui Chen, Shuang Xu, Cong‐Rui Liao, Anran Xu, Yue Han, Min‐Hui Yang, Li Zhao, Sha‐Sha Hu, Lan Wang, Qing‐Yuan Li, Ling‐Ying Zhan, Yan‐Qing Ding, Shuang Wang

**Affiliations:** ^1^ Department of Pathology Nanfang Hospital Southern Medical University Guangzhou Guangdong China; ^2^ Department of Pathology School of Basic Medical Sciences Southern Medical University Guangzhou Guangdong China; ^3^ Division of Spine Surgery Department of Orthopaedics Nanfang Hospital Southern Medical University Guangzhou Guangdong China; ^4^ Guangdong Provincial Key Laboratory of Gastroenterology Department of Gastroenterology Nanfang Hospital Southern Medical University Guangzhou Guangdong China

**Keywords:** colorectal carcinoma, epigenetic regulation, SEMA3B, *SEMA3B‐AS1*, tumor angiogenesis

## Abstract

Mounting evidence has demonstrated the considerable regulatory effects of long noncoding RNAs (lncRNAs) in the tumorigenesis and progression of various carcinomas. LncRNA Semaphorin 3B (SEMA3B) antisense RNA 1 (*SEMA3B‐AS1*) has been found to be dysregulated in a few carcinomas recently. However, its potential function and mechanism in colorectal carcinoma (CRC) have not yet been examined. Here we show that *SEMA3B‐AS1* acts as a crucial regulator of CRC progression. We found that *SEMA3B‐AS1* expression was downregulated in CRC cell lines and tissues. Downregulation of *SEMA3B‐AS1* was significantly associated with poor survival in CRC patients. Overexpression of *SEMA3B‐AS1* reduced the cell growth and metastasis of CRC in vivo and in vitro. In addition, *SEMA3B‐AS1* promoted the expression of its sense‐cognate gene SEMA3B, a member of the Semaphorin family (SEMAs), by recruiting EP300 to induce H3K9 acetylation at the SEMA3B promoter. Furthermore, we proved that *SEMA3B‐AS1* suppressed CRC angiogenesis by affecting the vascular endothelial growth factor signaling pathway activation which was regulated by the SEMA3B‐NRP1 axis. Our work unravels a novel mechanism of *SEMA3B‐AS1* in the inhibition of CRC malignant progression and highlights its probability as a new promising diagnostic marker and therapeutic target for CRC interventions.

## Graphical abstract

Schematic diagram showing the mechanism of action of *SEMA3B‐AS1* in CRC. *SEMA3B‐AS1* was downregulated in CRC and consequently decreased EP300 recruitment. And then, the reduction in EP300 recruitment suppressed the accumulation of the active marker H3K9ac and repressed SEMA3B levels. Subsequently, the receptor NRP1 interacted with less SEMA3B, and then the VEGF pathway was activated, which induced angiogenesis and CRC progression.

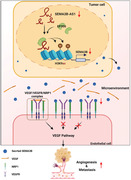

## 1 INTRODUCTION

Colorectal carcinoma (CRC) is the third most frequently occurring carcinoma, with over 1.9 million new cases and 0.935 million deaths all around the world in 2020.^1^ Surgery, radiotherapy, and chemotherapy are the main methods of treatment for CRC, but the death rate remains high, especially in patients with metastasis.^2^, ^3^ Therefore, a further comprehension of the molecular mechanisms of CRC onset and progression and the identification of novel biomarkers are crucial for improved diagnostic and treatment options.

Recently, novel biomarkers named long noncoding RNAs (lncRNAs) have been demonstrated that are abnormally expressed in many cancers^4^ and play significant roles in all kinds of cellular events, such as epigenetic gene regulation, transcriptional control, protein translation, and mRNA processing, eventually leading to tumor proliferation, metastasis, and recurrence.^5^, ^6^, ^7^, ^8^ Studies have revealed that dysregulated lncRNAs can lead to malignant evolution in CRC. For example, lncRNA SNHG29 inhibits the protein degradation of YAP mediated by phosphorylation and ubiquitination, thereby upregulating the PD‐L1 expression, resulting in CRC immune escape.^9^ Our previous study found that lncRNA SATB2‐AS1 suppressed CRC metastasis by upregulating SATB2 expression, inhibiting Snail transcription, and repressing epithelial‐to‐mesenchymal transition.^10^ However, many regulation mechanisms of lncRNAs on CRC progression still remain unclear. To explore novel lncRNAs, which may play key regulatory roles in CRC, we analyzed the lncRNAs differential expression profiles in CRC tissues and paired paracancerous normal intestinal mucosal tissues, as well as in CRC cells that derived from a primary tumor (SW480) and lymph node metastasis (SW620) from the same patient. We found that lncRNA Semaphorin 3B (SEMA3B) antisense RNA1 *(SEMA3B‐AS1)* was significantly downregulated in CRC.

Class 3 Semaphorins (SEMA3) are the only secreted proteins in SEMAs, which were first identified as repulsive molecules of neural axonal growth cone.^11^ SEMA3 also functions as tumor regulator by inhibiting or promoting cancer cell proliferation, metastasis, and angiogenesis.^12^ SEMA3B was described as a candidate suppressor in lung cancer, esophageal cancer, and prostate cancer^13^, ^14^, ^15^ and was transcribed head‐to‐head with *SEMA3B‐AS1*. Present improvements showed that *SEMA3B‐AS1* expression was decreased in triple‐negative breast cancer, gastric cardia adenocarcinoma, and esophageal squamous cell carcinoma,^16^, ^17^, ^18^ and low *SEMA3B‐AS1* expression levels were closely associated with poor survival in breast cancer and hepatocellular carcinoma.^19^, ^20^ However, lncRNAs affect gene and protein expression at transcriptional, epigenetic, and posttranscriptional levels.^21^ and play extremely important regulatory functions in tumorigenesis and tumor progression, such as leading to tumor angiogenesis and inducing immunosuppressive microenvironment.^22^, ^23^ Although *SEMA3B‐AS1* is dysregulated in some tumors, the expression, biological function, and mechanisms of *SEMA3B‐AS1* in CRC have not been reported so far.

In the current study, we demonstrated that *SEMA3B‐AS1* downregulation was connected with CRC patients’ poor prognosis, especially advanced‐stage patients. Enhancement of *SEMA3B‐AS1* expression resulted in suppression of CRC cells’ proliferation and metastasis in vitro and in vivo. We also showed that *SEMA3B‐AS1* regulated SEMA3B transcription by recruiting EP300 to promote the histone H3K9 acetylation of SEMA3B. Further exploration revealed that SEMA3B competitively inhibited vascular endothelial growth factor (VEGF) signaling pathway activation by binding to the NRP1 receptor, thus suppressing the invasion and metastasis of CRC. In summary, this research describes a novel *SEMA3B‐AS1*–EP300–SEMA3B–NRP1 axis and unravels the potential molecular mechanisms of lncRNA *SEMA3B‐AS1* in CRC progression, providing novel ideas for the diagnosis and treatment of CRC.

## 2 RESULTS

### 2.1 LncRNA *SEMA3B‐AS1* is significantly downregulated in CRC

**FIGURE 1:**
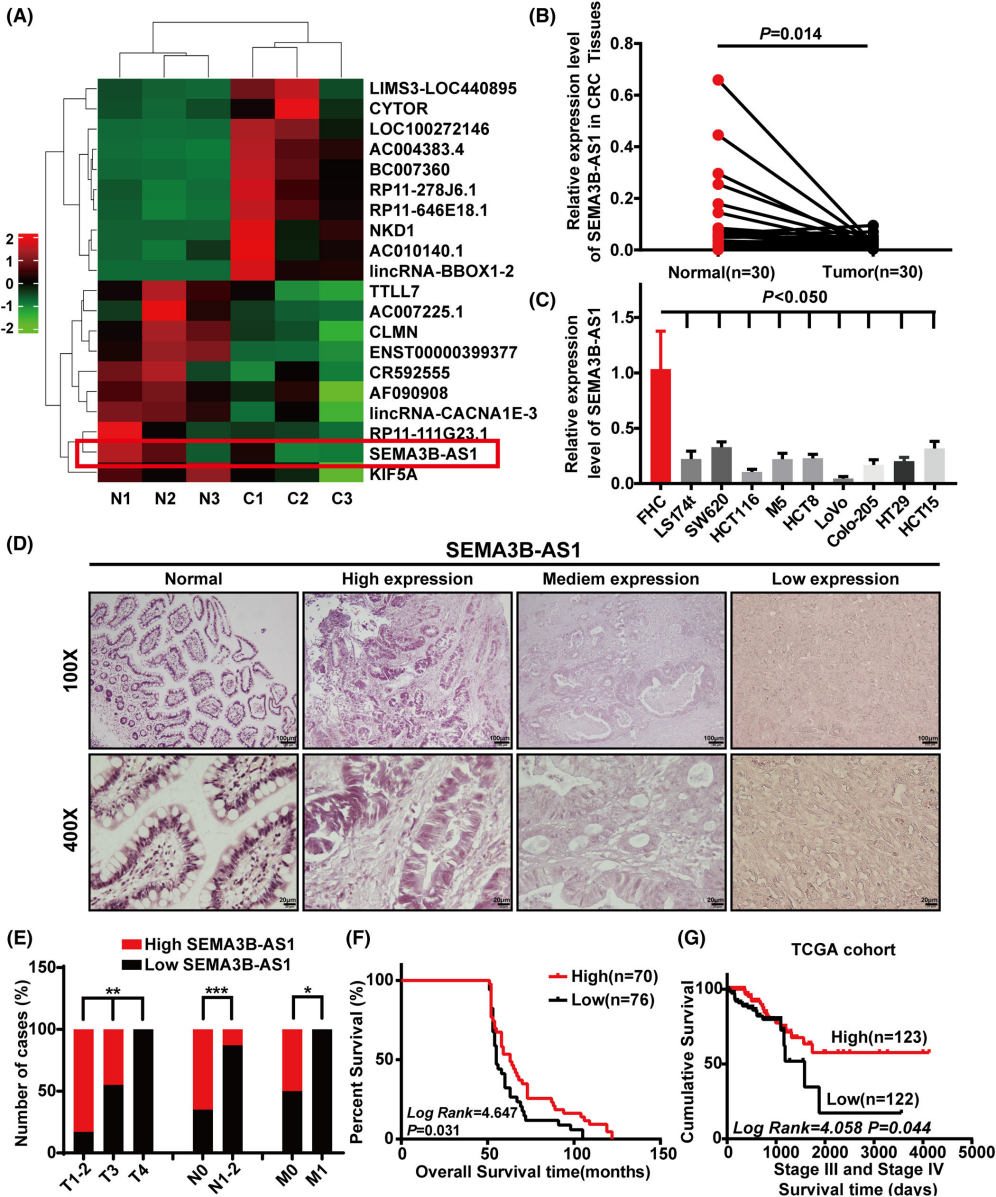
Long noncoding RNA (**LncRNA) *SEMA3B‐AS1* could be an important prognostic factor in predicting the survival of colorectal carcinoma (CRC) patients**. (A) Hierarchical clustering analysis of differentially expressed lncRNAs that intersected between CRC tissues and paracancerous normal intestinal mucosal tissues, as well as between CRC cells SW620 and SW480. N, normal intestinal mucosa tissues. C, colorectal carcinoma tissues. The red square highlights *SEMA3B‐AS1*. (B) The level of *SEMA3B‐AS1* in paired colorectal carcinoma and adjacent noncancerous tissues. (C) The level of *SEMA3B‐AS1* in colorectal carcinoma and colon mucosa epithelial (FHC) cell lines. (D) Expression analysis of *SEMA3B‐AS1* in normal colorectal mucosa and colorectal carcinoma tissues by in situ hybridization (ISH). Scale bars are shown in the right–left corner of each picture. The scale bar indicates 100 μm in upper row, and the scale bar indicates 20 μm in lower row. (E) Correlation among TNM, lymph node metastasis, distant metastasis, and expression of *SEMA3B‐AS1*. (F and G) Kaplan–Meier survival analysis in all patients with colorectal carcinoma according to *SEMA3B‐AS1* expression according to our data (F) and The Cancer Genome Atlas (TCGA) cohort (G). For (B)–(E), data are presented as means ± SD in three independent experiments. *p < 0.05; **p < 0.01; ***p < 0.001.

To explore the differentially expressed lncRNAs, which might be associated with CRC progression, we performed a lncRNA microarray, intersected the differentially expressed lncRNAs between CRC tissues and paracancerous normal intestinal mucosal tissues, as well as the differentially expressed lncRNAs between CRC cells derived from lymph node metastasis (SW620) and a primary tumor (SW480) from the same patient, and found 104 downregulated and 189 upregulated lncRNAs in CRC tissues. We closely paid attention to the top 10 lncRNAs in upregulated and downregulated sets. After removing the 12 lncRNAs that have not been included in NCBI and 5 lncRNAs that were eventually confirmed to be pseudogene or protein‐coding genes, we found that lncRNA *SEMA3B‐AS1* (ENST00000421735) had the highest change fold in the downregulated set (Figure 1A). To validate this finding, we first analyzed the expression of *SEMA3B‐AS1* in 30 pairs of fresh samples, including CRC tissues and matched adjacent normal intestinal epithelial tissues. The results revealed that the *SEMA3B‐AS1* expression was lower in CRC tissue samples than that in nontumor tissues from the same donor (*p* = 0.014, Figure 1B). Meanwhile, we observed a similar trend in two independent CRC samples (*p* < 0.001, Figure S1A) and in paired CRC samples (*p* *=* 0.004, Figure S1B) from The Cancer Genome Atlas (TCGA) cohort. Moreover, the *SEMA3B‐AS1* expression was much higher in colon mucosa epithelial cell line (FHC) than in nine CRC cell lines (Figure 1C). These results suggest that *SEMA3B‐AS1* is significantly downregulated in CRC.

### 2.2 Low *SEMA3B‐AS1* expression is relative to a poor prognosis in CRC patients

Additionally, in situ hybridization (ISH) was used to determine the quantity of *SEMA3B‐AS1* expression in an independent cohort of 146 primary CRC samples with extensive clinical follow‐up information. We found that *SEMA3B‐AS1* was mostly localized in the cell nucleus and was strongly expressed in normal tissues but markedly reduced in CRC tissues (Figure 1D). Then, all CRC patients were divided into low and high *SEMA3B‐AS1* expression groups in the light of the ISH staining score. The scoring criteria are detailed in the Supporting Information section. After analyzing the clinical data, we observed that lower *SEMA3B‐AS1* expression was related to advanced Tumour Node Metastasis (TNM) stage (*p* < 0.05, Figure 1E and Table S1). Survival analyses suggested that patients in low *SEMA3B‐AS1* expression group had poor survival than patients with high *SEMA3B‐AS1* levels (*p*  =  0.031, Figure 1F). The survival data in the TCGA cohort showed that low *SEMA3B‐AS1* expression had no effect on overall or early survival (*p* > 0.05, Figure S1C,D) but significantly contributed to a shorter survival time of advanced CRC patients (*p* = 0.044, Figure 1G). On the whole, these observations revealed that *SEMA3B‐AS1* was significantly associated with poor prognosis in advanced CRC patients.

### 2.3 *SEMA3B‐AS1* plays a crucial inhibitory role in proliferation and metastasis

**FIGURE 2:**
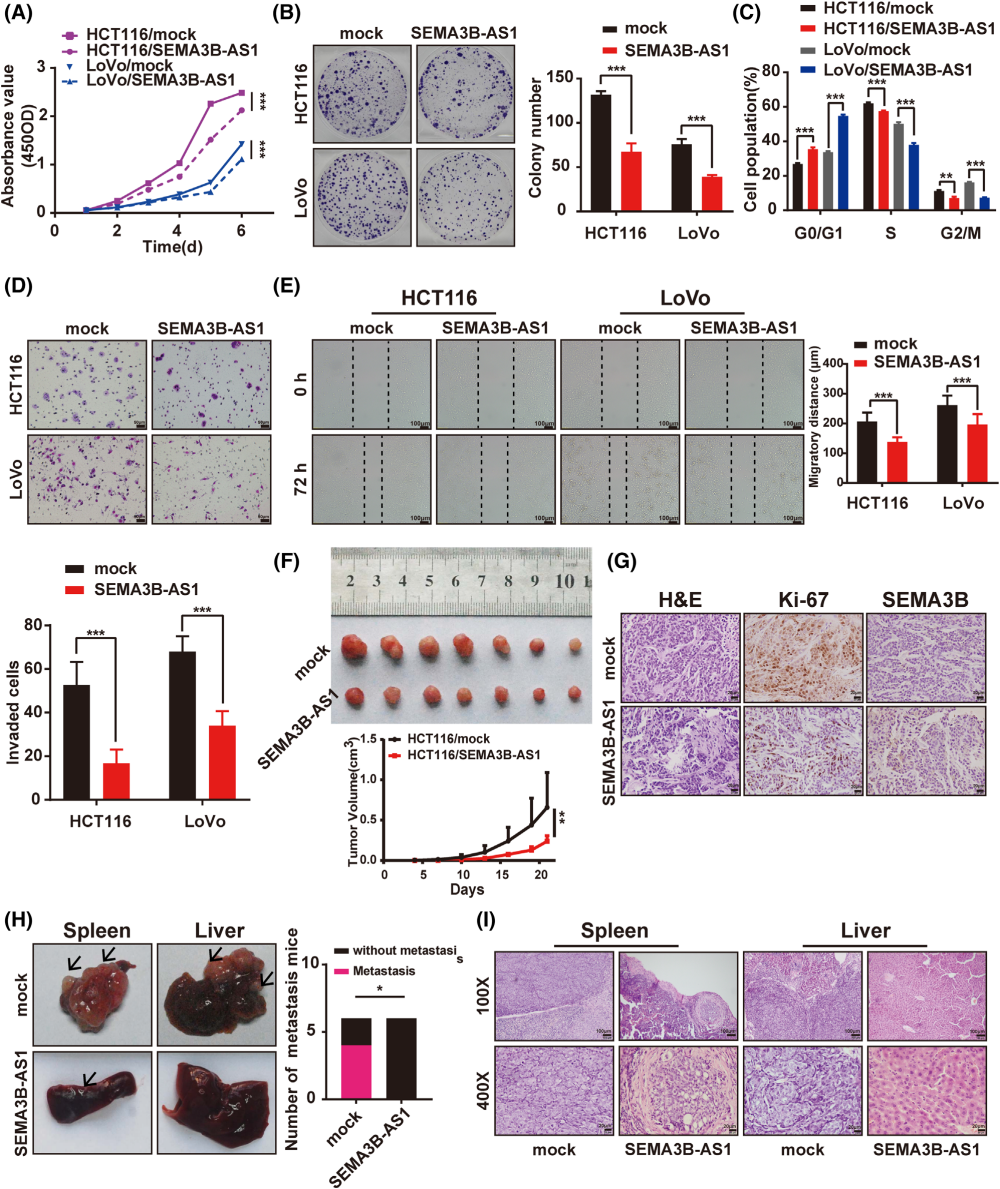
*SEMA3B‐AS1* overexpression inhibits colorectal carcinoma cell growth and metastasis in vitro and in vivo. (A) *SEMA3B‐AS1* overexpression suppressed cell proliferation in colorectal carcinoma cell lines as determined by CCK‐8 assay. (B) *SEMA3B‐AS1* overexpression inhibited colony formation in colorectal carcinoma cells. Representative images (left) and quantitative analyses (right) are shown. (C) *SEMA3B‐AS1* overexpression induced cell cycle arrest in the G1 phase in colorectal carcinoma cells. (D) *SEMA3B‐AS1* overexpression inhibited colorectal carcinoma cell invasion in a Matrigel invasion assay. Scale bars indicate 50 μm and are shown in the right–left corner of each picture. (E) *SEMA3B‐AS1* overexpression suppressed cell migration in the wound‐healing assay. The scale bar indicates 100 μm. The experiments were performed at least three times, and the data are expressed as the mean ± SD. (F) *SEMA3B‐AS1* overexpression inhibited subcutaneous tumor formation in nude mice. HCT116 cells with ectopic overexpression of *SEMA3B‐AS1* and control cells were inoculated into nude mice (*n* = 7 per group). The effect of *SEMA3B‐AS1* on colorectal carcinoma tumor growth was evaluated based on tumor volume in the two groups. (G) Representative photographs of hematoxylin and eosin (H&E) and immunohistochemistry (IHC) staining for Ki‐67 in primary cancer tissues. The scale bar indicates 20 μm. (H and I) Intrasplenic injections to establish a liver metastasis model in nude mice (*n* = 6 per group). The tumors in spleen and liver metastases after colorectal carcinoma (CRC) cell intrasplenic injections for 4 weeks (H, left) and the statistical distribution of metastasis numbers (H, right) are shown. The tissues were stained by H&E staining (I). The scale bar indicates 100 μm in upper row, and the scale bar indicates 20 μm in lower row. For (A)–(H), data are expressed as the means ± SD of three independent experiments. *p < 0.05; **p < 0.01; ***p < 0.001.

To explore the biological function of *SEMA3B‐AS1* in CRC, we established *SEMA3B‐AS1‐*stabilized overexpressed and *SEMA3B‐AS1*‐silenced CRC cell lines. The *SEMA3B‐AS1* expression was increased significantly in LoVo/*SEMA3B‐AS1* and HCT116/*SEMA3B‐AS1* cells and was significantly decreased in SW620‐si55, SW620‐si262, HCT15‐si55, and HCT15‐si262 cells compared with the mock or control group (*p* < 0.05, Figures S2 and S3A). Compared with the control cells, the *SEMA3B‐AS1* overexpression could markedly inhibit CRC cell growth showed by CCK‐8 assay (*p* < 0.001, Figure 2A). The capacity of the *SEMA3B‐AS1*‐overexpressing cells to form colonies was remarkably suppressed compared with that of the control cells (*p* < 0.001, Figure 2B). We investigated the underlying mechanism of the inhibition of *SEMA3B‐AS1* overexpression on proliferation and measured the cell cycle by flow cytometry. The data revealed that *SEMA3B‐AS1* overexpression could induce G1 phase arrest (*p* < 0.001, Figure 2C). Moreover, the wound‐healing and the transwell assays revealed that *SEMA3B‐AS1* overexpression markedly decreased the abilities of migration and invasion of CRC cells (*p* < 0.001, Figure 2D,E). Furthermore, the opposite effects were observed in *SEMA3B‐AS1*‐silenced cells (*p* < 0.05, Figure S3). These data suggested that *SEMA3B‐AS1* exerts a significant inhibitory effect on CRC cell growth, invasion, and migration in vitro.

To further verify the in vitro findings, we subcutaneously injected HCT116/*SEMA3B‐AS1* or HCT116/mock cells into nude mice for xenoplantation to determine the effects of *SEMA3B‐AS1* on proliferation in vivo. Mice injected with HCT116/*SEMA3B‐AS1* cells revealed significant tumor growth inhibition and a weaker staining intensity of Ki‐67 than those injected with HCT116/mock cells (*p* < 0.01, Figure 2F,G), which was consistent with the in vitro results. Next, we determined whether the overexpression of *SEMA3B‐AS1* could inhibit tumor metastasis in vivo. We established a metastatic mouse model by injecting HCT116/*SEMA3B‐AS1* or HCT116/mock cells under the splenic capsule. The mice were sacrificed after injection for 4 weeks, and their spleens and livers were observed and analyzed by immunohistochemistry (IHC). The results showed that compared with the control group (four out of six mice), the number of definite hepatic metastasis mice was decreased in *SEMA3B‐AS1* overexpression group (0 out of 6 mice) (*p* = 0.014, Figure 2H,I). These results indicate a crucial inhibitory effect of *SEMA3B‐AS1* on tumorigenesis and metastasis in vivo.

### 2.4 SEMA3B, the sense‐cognate gene for *SEMA3B‐AS1*, is regulated by *SEMA3B‐AS1* and acts as a tumor suppressor in CRC

**FIGURE 3:**
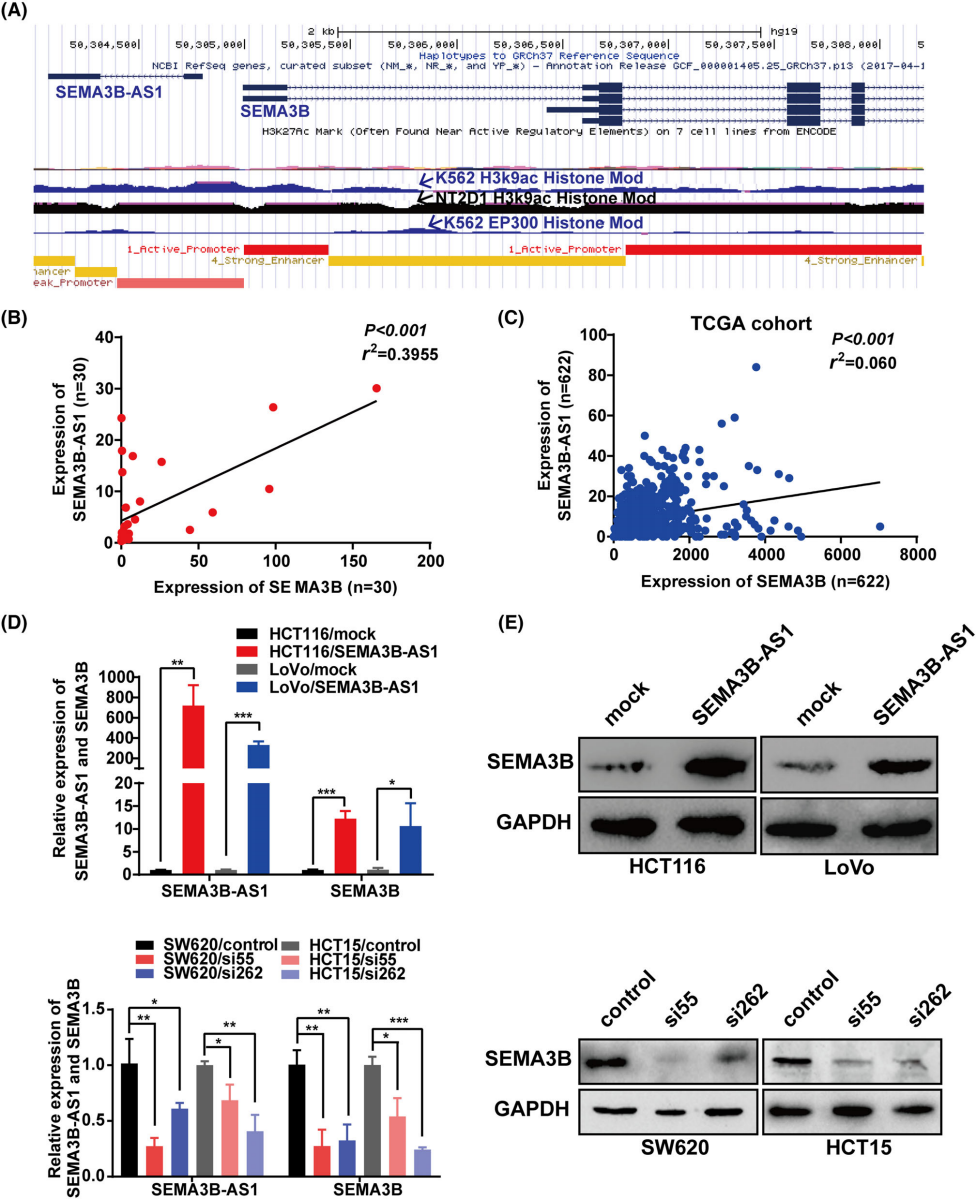
SEMA3B, the sense‐cognate gene for *SEMA3B‐AS1*, is a key downstream target of *SEMA3B‐AS1*. (A) Genomic location of SEMA3B and *SEMA3B‐AS1* from the ENCODE collection. Higher levels of epigenetic modification marks on histone 3 at lysine 9 (H3K9ac) and several transcription factor binding site uniform peaks of EP300 were observed within the SEMA3B promoter region. (B and C) The correlation between *SEMA3B‐AS1* transcript levels and SEMA3B mRNA levels in colorectal carcinoma tissues was measured according to our data (*n* = 30; B) and The Cancer Genome Atlas (TCGA) cohort (*n* = 622; C). (D and E) *SEMA3B‐AS1* regulated the expression of SEMA3B at the mRNA (D) and protein (E) levels. *p < 0.05; **p < 0.01; ***p < 0.001.

To explore the potential target genes of *SEMA3B‐AS1*, bioinformatics analysis was conducted using the UCSC Genome Browser website (http://genome.ucsc.edu/). Considering that *SEMA3B‐AS1* is the antisense lncRNA of SEMA3B and that the genomic locations of the two are very close (Figure 3A), we surmised that *SEMA3B‐AS1* might exert biological effects by regulating the expression of SEMA3B. Therefore, we measured the mRNA levels of SEMA3B in the same cohort as shown in Figure 1B. The results revealed that SEMA3B was downregulated in most CRC tissues significantly (*p* < 0.001, Figure S4B), and the RNA levels of *SEMA3B‐AS1* and SEMA3B in these samples were positively correlated (*r* ^2^ = 0.3599, *p* < 0.001, Figure 3B). A similar positive correlation was observed in the TCGA cohort (*n* = 622, *p* < 0.001, *r* ^2^ = 0.06, Figure 3C). Interestingly, we observed similar expression trends of *SEMA3B‐AS1* and SEMA3B mRNA levels in the same CRC cell line (Figure 1C and Figure S4A). Next, we measured the SEMA3B expression at mRNA and protein levels in the *SEMA3B‐AS1* overexpression or *SEMA3B‐AS1* knockdown CRC cells. Compared with the control cells, the mRNA and protein levels of SEMA3B were significantly increased in *SEMA3B‐AS1*‐overexpressed cells (*p* < 0.05, Figure 3D,E, upper) and significantly reduced in *SEMA3B‐AS1*‐knockdown cells (*p* < 0.05, Figure 3D,E, lower). Meanwhile, IHC detection of subcutaneous tumors in mouse models showed that *SEMA3B‐AS1* overexpression increase the protein levels’ expression of SEMA3B in vivo significantly (Figure 2G). Furthermore, we also observed that the expression changes of SEMA3B could affect the expression of *SEMA3B‐AS1* to a certain extent (*p* < 0.05, Figure S5). Overall, these observations suggest that *SEMA3B‐AS1* plays a key role in modulating its sense‐cognate gene SEMA3B expression.

In order to explore the expression and biological function of SEMA3B in CRC, we measured the expression of SEMA3B in CRC tissues and cells. The data indicated that the expressions of SEMA3B at protein and mRNA levels were significantly decreased in CRC cell lines and tissues (*p* < 0.05, Figure S4A–C). A similar conclusion was obtained by analyzing the TCGA cohort (*p* < 0.001, Figure S4D,E). The protein expression of SEMA3B in paraffin‐embedded tissue samples of CRC patients was also detected by IHC. The results showed that compared with the noncancerous samples, the CRC tissues displayed the lower expression of SEMA3B (Figure S4F).

We also constructed stable SEMA3B‐overexpressing and SEMA3B‐silenced CRC cells and performed functional experiments in vitro and in vivo. These results showed that SEMA3B overexpression suppressed cell growth and metastasis in vitro and in vivo (Figure S6) and that SEMA3B downregulation promoted CRC cell growth and metastasis in vitro (Figure S7). Overall, our data proved that SEMA3B is regulated by *SEMA3B‐AS1* and acts as a tumor suppressor in CRC.

### 2.5 *SEMA3B‐AS1* recruits EP300 to promote histone H3K9 acetylation of SEMA3B

**FIGURE 4:**
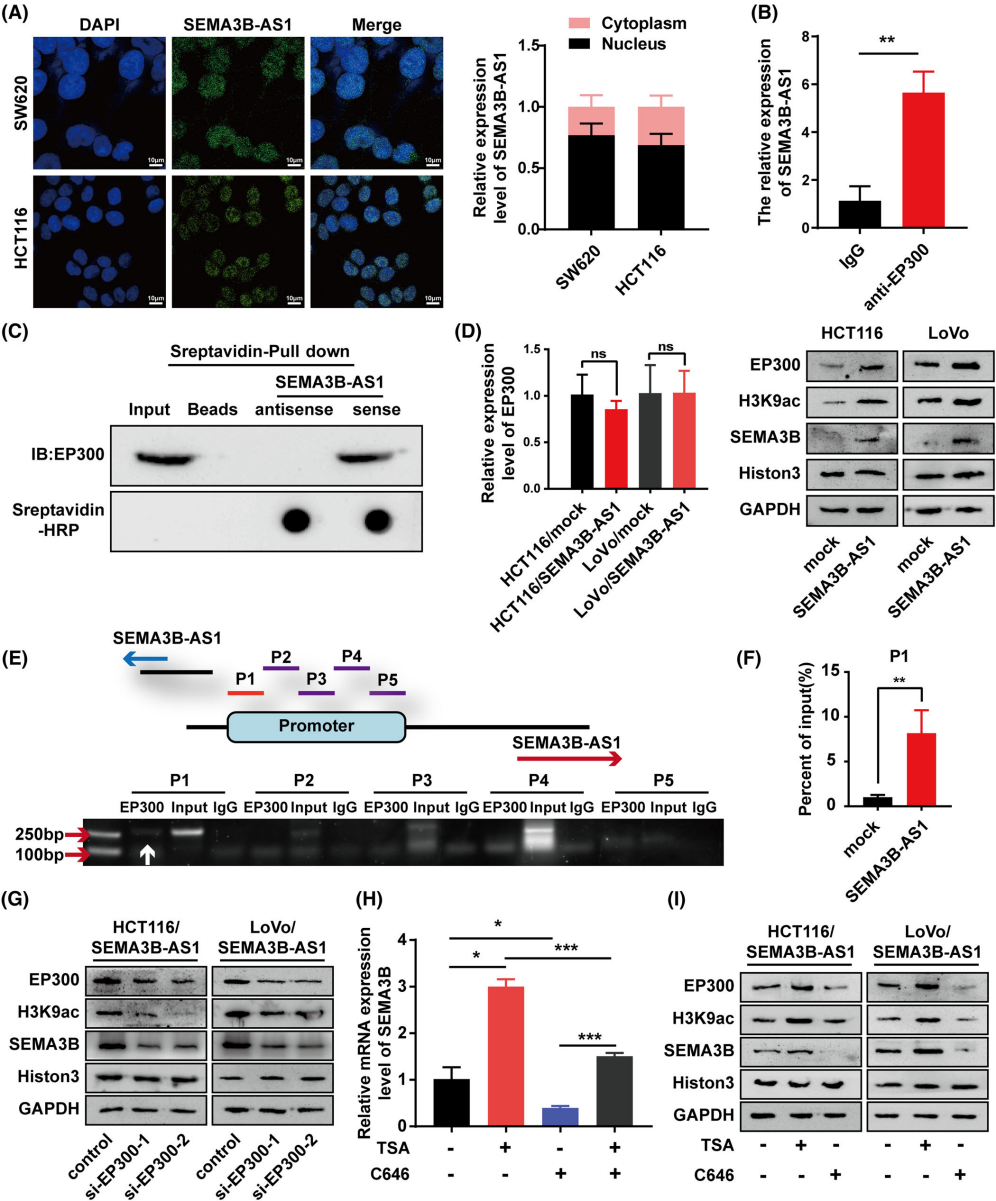
*SEMA3B‐AS1* promotes the expression of SEMA3B by recruiting EP300 to accelerate histone H3 acetylation. (A) Nuclear‐enriched *SEMA3B‐AS1* was determined by fluorescence in situ hybridization (left) and RNA fraction assays (right). (B) RIP experiments were performed in HCT116 cells using an EP300 or nonspecific IgG antibody to determine the amount of *SEMA3B‐AS1* RNA associated with EP300 or IgG relative to the input control. (C) RNA pull‐down assays were used to identify proteins associated with *SEMA3B‐AS1*. Biotinylated *SEMA3B‐AS1* and antisense RNA were incubated with cell extracts, and the associated proteins were resolved by SDS–PAGE. Western blotting was performed to analyze the specific interaction between EP300 and *SEMA3B‐AS1*. Dot blot of RNA–protein binding samples indicates equal RNA transcripts present in the assay. (D) *SEMA3B‐AS1* did not affect EP300 mRNA expression levels (left), but *SEMA3B‐AS1* overexpression significantly increased the protein levels of EP300, H3K9ac, and SEMA3B (right). (E and F) EP300 can bind to the SEMA3B promoter. A schematic illustration of the *SEMA3B‐AS1* and SEMA3B structures is shown (E, top). Arrows show the direction of transcription. The numbered sites denote the promoter fragments of the SEMA3B gene. The ability of EP300 to bind to SEMA3B promoter regions was assessed by chromatin immunoprecipitation (ChIP) (E) and was increased as a result of *SEMA3B‐AS1* overexpression (F). White arrow shows the SEMA3B promoter region that EP300 binds to (E). (G) Depletion of EP300 mediated by siRNA significantly decreased H3K9 acetylation and SEMA3B protein levels in colorectal carcinoma cell lines overexpressing *SEMA3B‐AS1*. (H) SEMA3B mRNA was regulated by the histone deacetylase inhibitor TSA and acetyltransferase inhibitor C646 in colorectal carcinoma cells. The C646 and TSA double‐negative group was set as control. (I) SEMA3B protein was regulated by the histone deacetylase inhibitor TSA and acetyltransferase inhibitor C646 in colorectal carcinoma cells. *p < 0.05; **p < 0.01; ***p < 0.001.

The function of lncRNAs is usually associated with their subcellular localization in cells.^24^, ^25^ Through fluorescence ISH experiment, we observed that *SEMA3B‐AS1* was mainly located in the nucleus (Figure 4A, left panel). We also isolated nuclear RNA and cytoplasmic RNA from CRC cells and analyzed them by quantitative real‐time PCR (qRT‐PCR) and found a similar subcellular location for *SEMA3B‐AS1* (Figure 4A, right panel). As the function of nuclear‐enriched lncRNAs is associated with transcriptional and epigenetic regulation, we speculated that the mechanism by which *SEMA3B‐AS1* upregulates SEMA3B expression might be through the modulation of chromatin modification and transcription factor recruitment.

Further analysis of the ENCODE data in the UCSC Genome Browser revealed that the presence of a higher level of acetylation modification marks on histone 3 at lysine 9 (H3K9ac) in the SEMA3B promoter region in K562 and NT2D1 cells, and that several active promoters produce uniform peaks of EP300, an important histone acetyltransferase, in K562 cells (Figure 3A). Next up, an RNA immunoprecipitation (RIP) assay was conducted to demonstrate a significant enrichment of *SEMA3B‐AS1* in the anti‐EP300‐antibody pull down compared with the nonspecific IgG antibody pull down (*p* = 0.0018, Figure 4B). The previous study showed that p300 could be recruited by ZEB1‐AS1 to ZEB1 promoter.^26^ Therefore, ZEB1‐AS1 was used as a positive control for the RIP assay (Figure S8). Moreover, we performed an RNA pull‐down assay to validate the physical association between EP300 and *SEMA3B‐AS1* in vitro (Figure 4C). In addition, we found that the mRNA expression level of EP300 did not be affected by *SEMA3B‐AS1* overexpression (Figure 4D, left), but the protein content of EP300 was increased by *SEMA3B‐AS1* overexpression (Figure 4D, right). Thereafter, a chromatin immunoprecipitation (ChIP) assay indicated that EP300 bound to the P1 segment of the SEMA3B promoter directly (Figure 4E), and *SEMA3B‐AS1* overexpression enhanced its binding capability (*p* = 0.0086, Figure 4F). Overall, these findings demonstrated that *SEMA3B‐AS1* acted as a scaffold and regulated SEMA3B expression by recruiting EP300 to the SEMA3B promoter. As shown in Figure 4D (right), the overexpression of *SEMA3B‐AS1* increased the levels of SEMA3B and H3K9ac. We considered whether the enhancement of H3K9ac caused by *SEMA3B‐AS1* was mediated by EP300. Accordingly, we transfected specific EP300 siRNAs into *SEMA3B‐AS1*‐overexpressed cells. The results showed that the protein expressions of SEMA3B and H3k9ac were significantly decreased in EP300 knockdown cells (Figure 4G). The tumor cells were also treated with the histone acetyltransferase inhibitor C646 (10 mmol/L) and deacetylase inhibitor trichostatin A (TSA; 100 nmol/L). As expected, the SEMA3B mRNA and protein levels were upregulated by TSA and downregulated by C646 (Figure 4H,I). Meanwhile, the H3k9ac protein levels and the accumulation of EP300 protein could also be regulated by TSA and C646 (Figure 4I). Taken together, these results demonstrate that the transcript activation of SEMA3B is dependent on *SEMA3B‐AS1* recruiting EP300, which induces the acetylation of H3K9 at the SEMA3B promoter region.

### 2.6 EP300/SEMA3B are required by *SEMA3B‐AS1* to inhibit CRC proliferation, invasion, and migration

**FIGURE 5:**
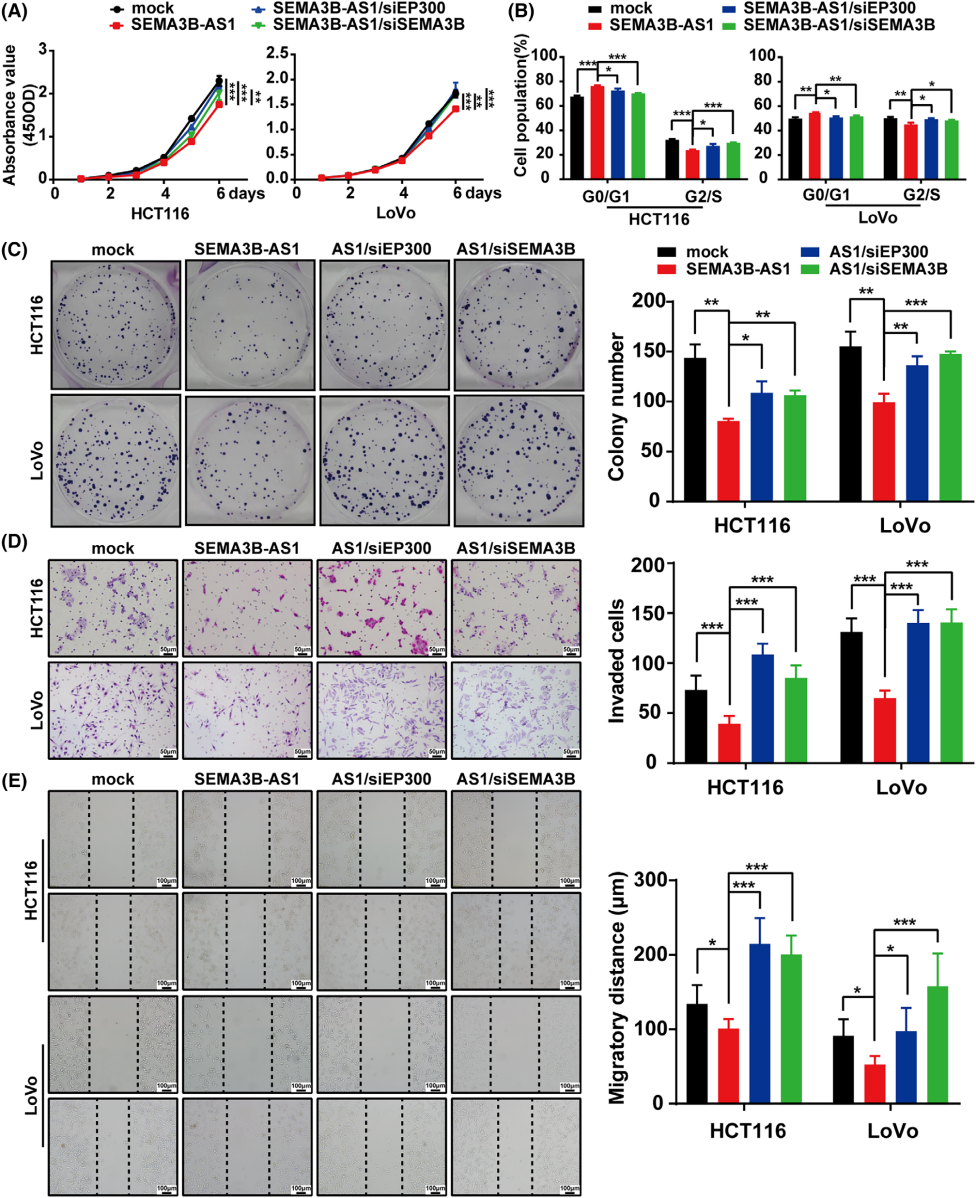
*SEMA3B‐AS1* requires EP300/SEMA3B to suppress colorectal carcinoma cell growth, invasion, and migration. *SEMA3B‐AS1* overexpression significantly reduced the colorectal carcinoma cell proliferation (A), cell cycle progression (B), colony formation (C), invasion (D), and migration (E). The potential effects of *SEMA3B‐AS1* on colorectal carcinoma cells were completely abolished, similar to the control cells, by EP300 knockdown by siRNA. The depletion of SEMA3B impairs colorectal carcinoma tumor cell proliferation efficiency and diminishes the suppressive effects of *SEMA3B‐AS1* overexpression in a similar manner. Scale bars indicate 50 μm (D) and 100 μm (E). *p < 0.05; **p < 0.01; ***p < 0.001.

To detect the contribution of EP300 and SEMA3B to the inhibitory effects of *SEMA3B‐AS1*, we knocked the EP300 or SEMA3B down in *SEMA3B‐AS1*‐overexpressed cells with specific siRNAs targeting EP300 or SEMA3B separately. The CCK‐8 assay displayed that CRC cells with *SEMA3B‐AS1* plus siRNA of EP300 or SEMA3B grew faster than *SEMA3B‐AS1* group (Figure 5A). Furthermore, the cell cycle distribution was analyzed by the flow cytometry, and results revealed that *SEMA3B‐AS1* combined with siRNA of EP300 or SEMA3B reversed *SEMA3B‐AS1*‐induced cell cycle arrest, resulting in more cells entering the G2/S phase (Figure 5B). The colony assay showed that compared with CRC cells overexpressing *SEMA3B‐AS1* alone, the group with *SEMA3B‐AS1* overexpression together with siRNA of EP300 or SEMA3B exerted more clones (Figure 5C). Likewise, the transwell assay and the wound‐healing assay showed that the suppression of invasion and migration induced by *SEMA3B‐AS1* overexpression was significantly reduced when EP300 or SEMA3B was knocked down (Figure 5D,E). These rescue effects suggested that the ability of *SEMA3B‐AS1* to inhibit proliferation, migration, and invasion is largely attributed to its ability to recruit EP300, which results in the activation of SEMA3B transcription.

### 2.7 *SEMA3B‐AS1* and SEMA3B inhibit angiogenesis through the VEGF signaling pathway

**FIGURE 6:**
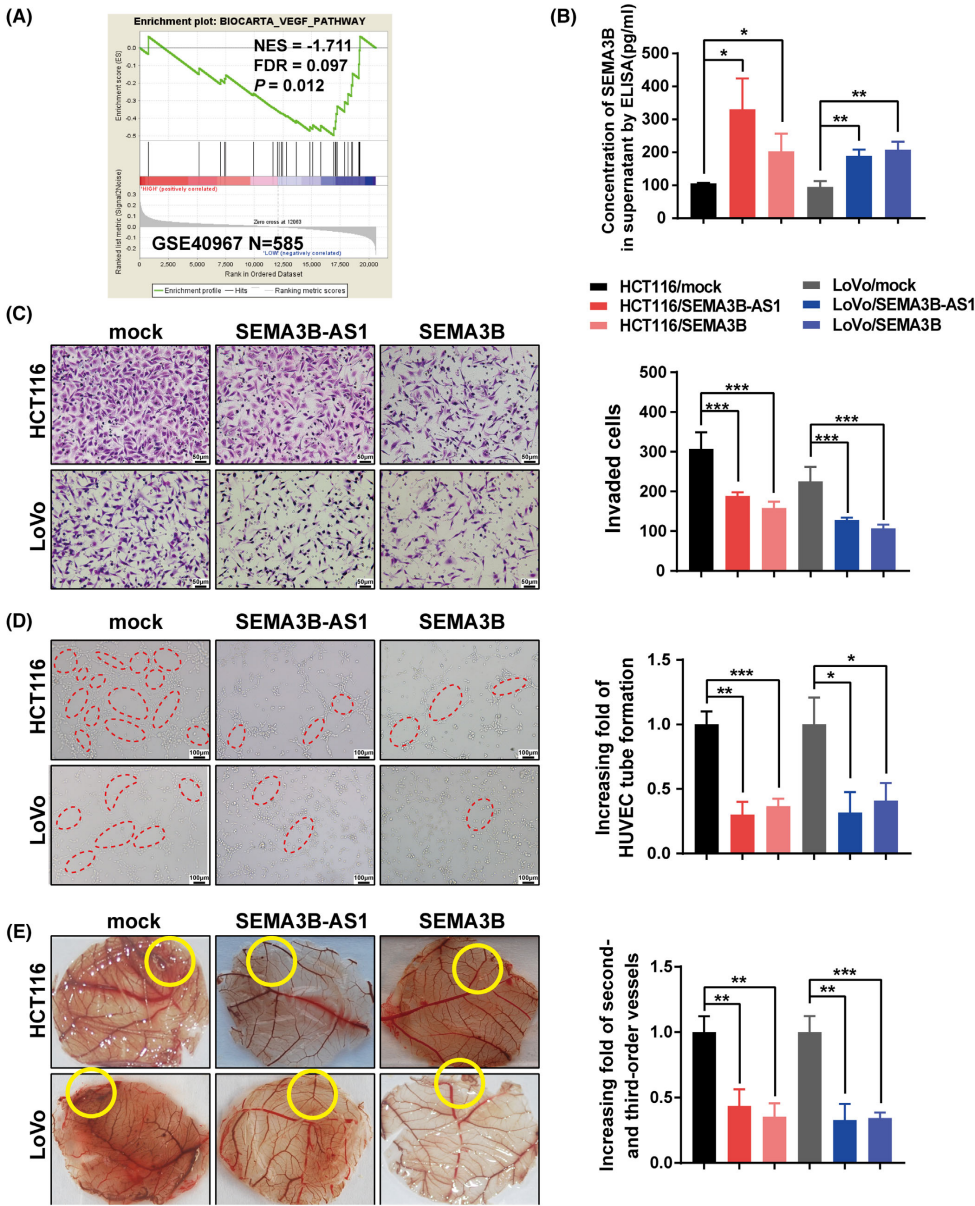
SEMA3B overexpression inhibits angiogenesis and the vascular endothelial growth factor (VEGF) pathway. (A) Gene set enrichment analysis (GSEA) showed the enrichment of the VEGF pathway in CRC cells with SEMA3B downregulation. (B) Overexpression of *SEMA3B‐AS1* or SEMA3B increased the content of SEMA3B protein in the supernatant of colorectal cancer cells. (C) Colorectal carcinoma (CRC) cell supernatant overexpressing *SEMA3B‐AS1* or SEMA3B inhibited the invasion ability of endothelial cells. Representative images (left) and the statistical analysis (right) are shown. Scale bars indicate 50 μm. (D) CRC cell supernatant overexpressing *SEMA3B‐AS1* or SEMA3B inhibited the tube formation of endothelial cells in vitro. Scale bars indicate 100 μm. (E) The chorioallantoic membrane (CAM) assay was used to examine blood vessel formation after stimulation with the supernatants from the indicated cells. The yellow circles indicate locations where conditioned medium was used. *p < 0.05; **p < 0.01; ***p < 0.001.

Gene set enrichment analysis of SEMA3B‐regulated gene signatures showed that SEMA3B expression was negatively correlated with VEGF signaling pathway enrichment (GSE40967, Figure 6A). We hypothesized that SEMA3B secreted by CRC cells might regulate tumor angiogenesis. We first found that the content of SEMA3B increased in the supernatant of *SEMA3B‐AS1*‐overexpressing and SEMA3B‐overexpressing cells (*p* < 0.05, Figure 6B). Then, we performed a transwell assay to assess the effects of secreted SEMA3B on human umbilical vein endothelial cell (HUVECs) migration. We observed that the supernatant of *SEMA3B‐AS1*‐overexpressing and SEMA3B‐overexpressing cells decreased the migration of HUVECs significantly (*p* < 0.001, Figure 6C). Furthermore, we found that *SEMA3B‐AS1*‐overexpressing and SEMA3B‐overexpressing culture media both strongly inhibited the HUVEC tubule formation in vitro and inhibited angiogenesis in chorioallantoic membrane (CAM) in vivo by tubule formation and chicken CAM assays (*p* < 0.05, Figure 6D,E). These observations suggest that the secretion of SEMA3B induced by *SEMA3B‐AS1* inhibits tumor angiogenesis via the VEGF signaling pathway.

### 2.8 The inhibitory effect of *SEMA3B‐AS1* on tumor angiogenesis depends on the competitive combination of SEMA3B and VEGF with NRP1

**FIGURE 7:**
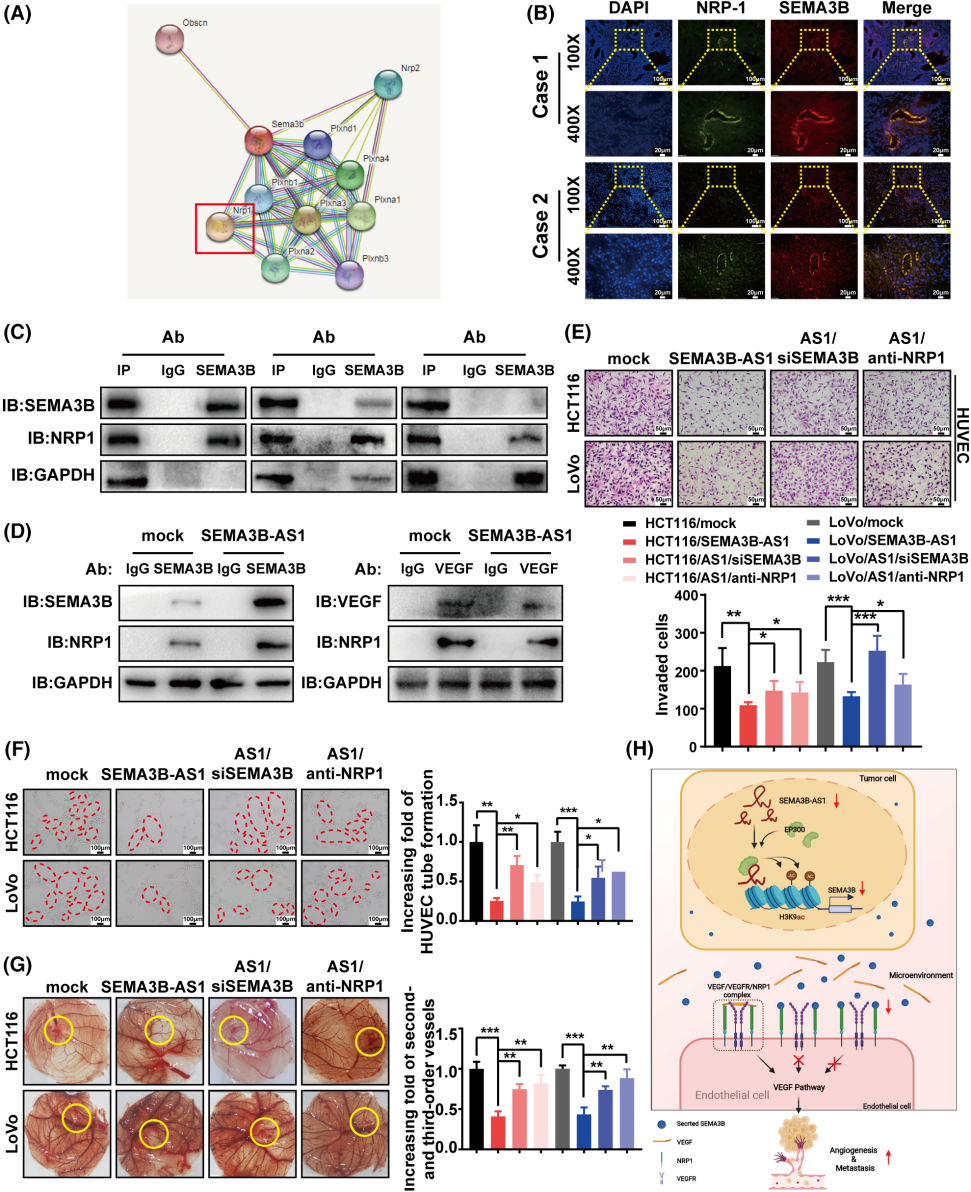
*SEMA3B‐AS1* requires SEMA3B/NRP1 to suppress angiogenesis. (A) Protein network analysis of possible SEMA3B interacted proteins. The red square highlights NRP1. (B) SEMA3B and NRP1 colocalize in the blood vessels of colorectal carcinoma (CRC) patients. The scale bars indicate 100 and 20 μm in pictures at 100× or 400× magnifications, respectively. (C) Both SEMA3B and vascular endothelial growth factor (VEGF) interact with NRP1, as analyzed by coIP. (D) NRP1 can bind with more SEMA3B and less VEGF after *SEMA3B‐AS1* overexpression. (E–G) *SEMA3B‐AS1* required SEMA3B/NRP1 to suppress the human umbilical vein endothelial cell (HUVEC) invasion (E), tubule formation in vitro (F), and angiogenesis in the chorioallantoic membrane (CAM) assay (G). Scale bars indicate 50 μm (E) and 100 μm (F), respectively. The yellow circles in (G) indicate locations where conditioned medium was used. (H) Schematic diagram showing the mechanism of action of *SEMA3B‐AS1* in CRC. *SEMA3B‐AS1* was downregulated in colorectal carcinoma and consequently decreased EP300 recruitment. In turn, the reduction in EP300 recruitment suppressed the accumulation of the active marker H3K9ac and repressed SEMA3B levels. Subsequently, the receptor NRP1 interacted with less SEMA3B, and then, the VEGF pathway was activated, which induced angiogenesis and colorectal carcinoma progression.*p < 0.05; **p < 0.01; ***p < 0.001. *Source*: Illustration created with BioRender (available online: https://biorender.com/ (accessed on 15 June 2022)).

A network analysis of potential interacting proteins of SEMA3B showed that SEMA3B might interact with neuropilin‐1 (NRP1) (Figure 7A). Previous studies have shown that NRP1 is widely expressed on the surface of vascular endothelial cells, acting as a coreceptor of VEGF, forming complexes with VEGF receptors (VEGFRs) to enhance the interaction between VEGFRs and VEGF, thus promoting tumor metastasis.^29^ Immunofluorescence revealed that SEMA3B and NRP1 colocalized on the vascular endothelium of CRC tissues (Figure 7B). Coimmunoprecipitation assays further indicated that both SEMA3B and VEGF could bind to NRP1, but there was no interaction between SEMA3B and VEGF (Figure 7C). Notably, compared with the control group, NRP1 could bind with more SEMA3B and less VEGF in *SEMA3B‐AS1*‐overexpressing cells (Figure 7D), and the content of SEMA3B increased in the cell culture supernatant with increasing exogenous VEGF concentration (Figure S9). These results suggested that SEMA3B competitively binds NRP1 with VEGF to regulate the activation of the VEGF signaling pathway and inhibit tumor angiogenesis.

Finally, we knocked down SEMA3B or blocked NRP1 on the basis of overexpression of *SEMA3B‐AS1* and carried out rescue experiments using the above conditioned media. We found that the inhibition of endothelial cell invasion and tubule formation in vitro and antiangiogenesis in vivo induced by the overexpression of *SEMA3B‐AS1* were recovered to a certain extent after the expression of SEMA3B and NRP1 was interfered (*p* < 0.05, Figure 7E–G). Collectively, these findings indicate that *SEMA3B‐AS1* suppresses CRC angiogenesis by affecting the activation of the VEGF signaling pathway, which is mediated by the SEMA3B–NRP1 axis (Figure 7H).

## 3 DISCUSSION

In present study, we discovered lncRNA *SEMA3B‐AS1*, the expression levels of which were downregulated in CRC tissues significantly, and the low expression of *SEMA3B‐AS1* was closely associated with TNM staging and poor prognosis in advanced CRC patients. We also proved that *SEMA3B‐AS1* functioned as a suppressor during CRC tumorigenesis and progression. Besides, we proved that *SEMA3B‐AS1* accelerated the transcriptional activation of its sense‐cognate gene SEMA3B, which also played an important inhibitory role in CRC progression by recruiting EP300 to the SEMA3B promoter and increasing acetylation levels of H3K9 at the SEMA3B promoter region. Then, SEMA3B was secreted into the tumor microenvironment and competitively bound to the NRP1 receptor on vascular endothelial cells with VEGF to suppress the VEGF signaling pathway activation and to ultimately suppress tumor angiogenesis. Thus, our results reveal a new *SEMA3B‐AS1*–SEMA3B–NRP1 axis, which is involved in epigenetic regulation and tumor microenvironment regulation.

With the development of novel sequencing technologies, it became clear that lncRNAs may potentially be very valuable novel cancer biomarkers, which regulate gene expression at the transcriptional, epigenetic, and posttranscriptional levels.^27^ We focused on lncRNA *SEMA3B‐AS1*, which is related to CRC progression processes, by high‐throughput lncRNA expression profiling. To date, only a few studies on *SEMA3B‐AS1* have been reported. For example, *SEMA3B‐AS1* and SEMA3B function synergistically to suppress the progression of gastric cardia carcinoma and esophageal squamous cell carcinoma.^16^, ^17^ However, the biological effect and molecular mechanism of *SEMA3B‐AS1* in CRC progression remain unclear. Our results demonstrated that *SEMA3B‐AS1* was downregulated in CRC cell lines and tissues, and that low *SEMA3B‐AS1* expression was connected with TNM stages and poor prognosis of advanced CRC patients significantly. These data indicate that *SEMA3B‐AS1* may be a potential biomarker for predicting CRC prognosis.

Functionally, we found that *SEMA3B‐AS1* inhibited the proliferation and metastasis of CRC in vivo and in vitro. A study published during the course of our study showed that *SEMA3B‐AS1* overexpression could downregulate the cyclin D1 expression.^28^ Zhong's group found that *SEMA3B‐AS1* indirectly regulated PTEN to suppress the proliferation of hepatocellular carcinoma cells.^19^ The previous studies have reported that PTEN could regulate the cell cycle through a variety of pathways, such as downregulating cyclin D1,^29^ promoting p53 acetylation,^30^ and forming a complex with CDK1 and cyclin B1.^31^ Given our results and the studies described above, we considered that the inhibitory effects of *SEMA3B‐AS1* on CRC proliferation were mainly mediated by repressing cell cycle progression. However, the other potential mechanisms of CRC proliferation are worth further exploration in the future.

Antisense lncRNAs are transcribed from the opposite strand of noncoding or protein‐coding genes.^32^ Growing evidences demonstrated that antisense lncRNAs may regulate their sense‐cognate expression.^21^, ^33^, ^34^ For example, AFAP1‐AS1 promoted the expression of the AFAP1 protein in lung cancer.^35^ ZEB1‐AS1 activated the expression of ZEB1 in prostate cancer.^36^ SEMA3B, the sense‐cognate gene for *SEMA3B‐AS1*, belongs to the SEMAs and is involved in different biological processes, such as apoptosis, cell invasion, cell migration, and angiogenesis.^37^ In our study, we found that the RNA levels of *SEMA3B‐AS1* and SEMA3B in CRC were significantly correlated. Notably, a change in *SEMA3B‐AS1* abundance through overexpression and knockdown particularly affected SEMA3B mRNA and protein expression. SEMA3B is downregulated in many epithelial tumors, such as glioma^13^ and esophageal squamous cell carcinoma,^15^ and could act as a prognostic index reflecting the immune infiltration of breast cancer patients.^38^ Although Pronina et al. showed that the mRNA quantity of SEMA3B in CRC decreases frequently,^39^ the biological effects and molecular mechanisms of SEMA3B in CRC remain unclear. Our study indicated that SEMA3B was downregulated in CRC cells and tissues and inhibited CRC cell proliferation, invasion, and migration in vitro and in vivo. Thus, we speculated that *SEMA3B‐AS1* positively regulates SEMA3B expression and that the two suppress CRC progression synergistically.

The subcellular localization of lncRNAs usually determines their function.^24^, ^25^ A vast number of nuclear‐enriched lncRNAs have been proven to modify gene expression in cancer by taking advantage of epigenetic programs. For example, in CRC, HOXD‐AS1 inversely regulated HOXD3 expression by binding with the PRC2 complex, which accumulated H3K27 trimethylation at the HOXD3 promoter region.^33^ Our previous study showed that SATB2‐AS1 modulated CRC aggressiveness by the epigenetic regulation of SATB2 and Snail expression.^10^ In this article, we proved that *SEMA3B‐AS1* was enriched in the nucleus, suggesting its potential for epigenetic regulation. Guo et al. found that *SEMA3B‐AS1* interacted with MLL4, an important enzyme that modifies H3K4 methylation, to induce the expression of SEMA3B.^17^ They also showed an enrichment of H3K9ac within the SEMA3B promoter region in BGC‐823 cells,^17^ but the mechanism that caused this phenomenon was not clarified. Our findings showed that *SEMA3B‐AS1* could recruit and bind to the lysine acetyltransferase EP300, thereby inducing the accumulation of H3K9ac within the SEMA3B promoter, which activates SEMA3B transcription. Moreover, the suppression induced by *SEMA3B‐AS1* on cell growth, migration, and invasion was partially reversed by the knockdown of SEMA3B or EP300. Hence, our data indicate a novel mechanism, in which *SEMA3B‐AS1* promotes SEMA3B expression through epigenetic regulation and suggest a crucial role of the *SEMA3B‐AS1*/EP300/SEMA3B pathway in CRC progression. However, lncRNAs could regulate the expression of genes through various mechanisms except acting as “scaffold.” For example, *SEMA3B‐AS1* could act as “miRNA sponge” of miR‐3940‐3p to prevent the degradation of KLLN.^18^ Therefore, *SEMA3B‐AS1* might regulate multiple genes in different ways. Further research studies are needed to reveal the potential mechanisms.

In recent years, a growing number of evidence suggest that SEMAs and their receptors may play a crucial regulatory role in tumorigenesis and tumor progression. Studies showed that SEMAs could perform varied functions by binding to different receptors in different conditions. For instance, SEMA4A binds to Tim‐2 to activate T‐cell.^40^ SEMA4D binds to CD72 to promote the activity of CD8^+^ T‐cells in non‐small cell lung cancer patients.^41^ NRP1 is the classical receptor of the SEMAs.^42^ It is shown that SEMA3A interacted with NRP1 to mediate MelCAM expression, leading to the suppression of breast cancer cell growth and angiogenesis.^43^ SEMA3F binds to NRP1 to inhibit the small cell lung carcinoma cell colony formation.^44^ But SEMA3C binds to NRP1 to promote prostate cancer cell perineural invasion by activating cMET.^45^ These studies suggested that different members of SEMAs played diverse or even opposite roles through various mechanisms in different tumors. In CRC, whether SEMA3B interacts with NRP1 and its specific mechanism remain unclear. In this research, we showed that SEMA3B binds to NRP1 in CRC. Interestingly, SEMA3B is known as an inhibitor of angiogenesis in hepatocellular carcinoma.^46^ But Rolny et al. observed that SEMA3B potentiated tumor metastasis as well as tumor angiogenesis by NRP1‐dependent manner in many types of tumors, such as melanomas, gastric cancer, and lung cancer.^47^ Our results have demonstrated functional competition between SEMA3B and VEGF for NRP1 binding, thereby suppressing the activation of the VEGF signaling pathway and ultimately inhibiting tumor angiogenesis. Moreover, we observed that the inhibitory effects of endothelial cell invasion, tubule formation, and angiogenesis induced by *SEMA3B‐AS1* were recovered to some extent after the expression of SEMA3B was knocked down or NRP1 was blocked. In summary, our data reveal that the inhibitory effects of *SEMA3B‐AS1* on tumor angiogenesis depend on the SEMA3B–NRP1–VEGF pathway axis.

The progress of antiangiogenic therapy for CRC and improvements of its efficacy have always been the focus of basic and clinical research. Although VEGF pathway inhibitors were reported to improve survival in most patients with cancer, some patients experience little to no beneficial effects due to primary or acquired resistance.^48^ In a future study, we will focus on whether secreted SEMA3B combined with VEGF pathway inhibitors can effectively improve the therapeutic effect on CRC. In addition, tumor vessels are structurally and functionally abnormal.^49^ Notably, after antiangiogenic therapy, vascular structures and tumor vessel functions are transiently normalized.^50^ Consequently, more efficient drugs can be delivered into the tumor through tumor vascular normalization. It has been reported that SEMA3G is involved in modulating physiological vascular remodeling for reparative vascular regeneration and normalization.^51^ Therefore, whether *SEMA3B‐AS1* and SEMA3B can induce CRC vascular normalization while inhibiting tumor angiogenesis is an interesting issue for further investigation.

Increasing numbers of research studies showed that lncRNAs were involved in the regulation of tumor metastasis. However, many regulation mechanisms of lncRNAs on CRC metastasis still remain unclear. Our previous study revealed that SATB2‐AS1 inhibited EMT to suppress CRC metastasis by inhibiting SATB2‐dependent snail transcription.^10^ In this study, we verified by bioinformatics analysis and subsequent experiments that lncRNA *SEMA3B‐AS1* inhibited tumor angiogenesis by epigenetic regulating the expression of its sense gene SEMA3B, which further highlighted the importance of lncRNAs in epigenetic regulation and expanded the molecular mechanisms of CRC metastasis effected by lncRNAs.

## 4 CONCLUSIONS

In summary, we demonstrated that lncRNA *SEMA3B‐AS1* acts as an epigenetic regulator that recruits EP300, increases the accumulation of H3K9ac at the SEMA3B promoter region, and upregulates the expression of SEMA3B. Then, we showed that secreted SEMA3B inhibits tumor angiogenesis by competitively binding to NRP1 with VEGF and ultimately suppresses the proliferation, migration, and invasion of CRC cells. Overall, we established the previously unknown function and mechanism of *SEMA3B‐AS1* in CRC and highlighted the importance of SEMA3B‐AS1 in the tumorigenesis and progression of CRC. Our data also implicate *SEMA3B‐AS1* as a potential novel target for antiangiogenic therapies for CRC.

## 5 MATERIALS AND METHODS

### 5.1 Microarray and computational analysis

Three CRC tissues, the matched paracancerous normal intestinal mucosal tissues, and CRC cells derived from a primary tumor (SW480) and lymph node metastasis (SW620) from the same patient were used for the lncRNA expression profiling analysis. In brief, we first extracted the total RNA CRC tissues and cells. Then, the RNA was synthesized into double‐stranded cDNA. LncRNA Expression Microarray (Arraystar) was used to label, purify, and hybridize the cDNA. An Agilent Microarray Scanner (Agilent p/n G2565BA) was used to scan slides after hybridization and washing. Finally, we extracted the data using Agilent Feature Extraction Software. We use Student's *t* test to calculate a *p* value. The differentially expressed genes threshold was set as a *p* value ≤0.05 and a fold change ≥2.0. The microarray data in this study have been uploaded to the National Center for Biotechnology Information (NCBI) Gene Expression Omnibus (GEO). The GEO Series accession number is GSE184903.

### 5.2 Clinical specimens

A total of 146 paraffin‐embedded specimens were obtained from CRC patients from 2000 to 2005 at the Department of Pathology, Nanfang Hospital Southern Medical University (Guangzhou, China). The information of these patients, such as age, gender, tumor differentiation, tumor size, T stage, distant metastasis, and lymph node metastasis, were provided by the medical records. Patients were followed for a period ranging from 1 to 122 months. The specimens were used to investigate the *SEMA3B‐AS1* expression by ISH. Thirty pairs of fresh CRC tissue from patients and their matched paracancerous normal intestinal mucosal tissues, which were stored in liquid nitrogen prior to qRT‐PCR and western blotting analyses, were collected from Nanfang Hospital (Guangzhou, China). The patients had not received chemotherapy or radiotherapy before surgery. All patients provided their signed, informed consent prior to the collection of clinical materials. Nanfang Hospital's Protection of Human Subjects Committee approved the protocols followed in this article.

### 5.3 Cell cultures

The human CRC cell lines HCT15, HCT116, SW620, LS174t, LoVo, HCT8, Colo‐205, HT29, and the FHC cell were purchased from the American Type Culture Collection (ATCC). The M5 cell, the ability of liver metastasis of which was enhanced, was obtained by the in vivo selection of SW480 from our previous study.^52^ The FHC cell and HUVECs were purchased from the cell bank at the Chinese Academy of Sciences (Shanghai, China). FHC, LS174t, SW620, HCT116, HCT8, LoVo, Colo‐205, HT29, HCT15, and HUVECs cells were cultured in RPMI 1640 or DMEM medium (Gibco) with 10% fetal bovine serum (Gibco BRL). They were maintained at 37°C in a humidified 5% CO_2_ atmosphere.

### 5.4 RNA isolation, reverse transcription (RT), and qRT‐PCR

AG RNAex Pro reagent (AG21102, Accruate Biotechnology) was used to extract total RNA from cells, tumor tissues, and normal intestinal mucosal tissues according to the instructions of manufacturer. The PrimeScript RT reagent Kit (RR047A, Takara) was used to synthesize cDNA. Three‐step qRT‐PCR was performed at least three times to take the assessment of technical variability into consideration using SYBR Premix Ex Taq (DRR041A, Takara) and the ABI PRISM 7500 Sequence Detection System (Applied Biosystems). The data were normalized to the expression of glyceraldehyde‐3‐phosphate dehydrogenase (GAPDH) and calculated with the 2^−ΔΔ^ *^CT^* method. The specific sequences of primers for amplification are presented in Table S2.

### 5.5 ISH and evaluation of *SEMA3B‐AS1* staining

ISH was performed according to the protocol of manufacturer (Exiqon). The sequence of 5′‐digoxigenin (DIG)‐labeled oligonucleotide probe of *SEMA3B‐AS1* is 5′‐TGGAGGGAGAGGTTGAGATATT‐3′. Further details are provided in the Supporting Information section.

### 5.6 Construction of stable overexpression of *SEMA3B‐AS1* and SEMA3B cell lines

The full‐length 357 bp DNA sequence of *SEMA3B‐AS1* and the full‐length 2247 bp DNA sequence of SEMA3B were amplified and cloned into the pLenti‐EF1a‐EGFP‐F2A‐Puro‐CMV‐MCS vector (OBiO Technology). Then, we transfected pLenti‐EF1a‐EGFP‐F2A‐Puro‐CMV‐MCS‐*SEMA3B‐AS1* or pLenti‐EF1a‐EGFP‐F2A‐Puro‐CMV‐MCS‐SEMA3B into HCT116 and LoVo cell lines with Lipofectamine 3000 reagent (Thermo Fisher Scientific) according to the instruction of manufacturer. The empty lentiviral vector pLenti‐EF1a‐EGFP‐F2A‐Puro‐CMV‐MCS was used as control. Transduced cells were selected in medium containing 3 μg/mL puromycin Sigma) and maintained in RPMI 1640 medium containing 1 μg/mL puromycin.

### 5.7 Oligonucleotide transfection

The siRNAs *SEMA3B‐AS1*, *SEMA3B*, *EP300*, and negative control were obtained from GenePharma. Oligonucleotide transfection was performed with Lipofectamine 3000 according to the instructions of manufacturer. The specific siRNAs’ sequences are shown in Table S3.

### 5.8 Functional assays in vitro

Cell in vitro proliferation, flow cytometry, wound‐healing, colony formation, and invasion assays were performed with the previously described methods.^10^ Further details are shown in the Supporting Information section. At least three times independent repeated experiments were conducted for all the above experiments.

### 5.9 Tumorigenic and metastasis assays in vivo

Male BALB/C‐nu/nu nude mice which were 4–6‐week old were obtained from the Laboratory Animal Centre of Southern Medical University. We provided further details in the Supporting Information section. All mice were maintained in a certain pathogen‐free facility and handled using the most humane practices.

### 5.10 Immunohistochemistry (IHC)

We performed IHC experiment as described previously^33^ with the following modifications. The slides were incubated at 4°C for 8 h with primary antibodies against SEMA3B (1:100, NB100‐2218SS, Novusbio) and Ki‐67 (1:500, 27309‐1‐AP, ProteinTech Group). The evaluation method for IHC staining was the same as that for ISH.

### 5.11 Western blotting analysis

The protocol of western blotting analysis and the details of the antibodies used are provided in the Supporting Information section.

### 5.12 Chromatin immunoprecipitation (ChIP) assay, RNA pull‐down assays, RNA immunoprecipitation (RIP) assay, and immunofluorescence

ChIP assay, RNA pull‐down assays, RIP assay, and immunofluorescence were performed as described previously.^10^ Further details are provided in the Supporting Information section. The specific PCR primers used for ChIP assays are listed in Table S2.

### 5.13 Chicken chorioallantoic membrane (CAM) assay

The sixth day of fertilized chicken eggs (Yueqin Breeding Co. Ltd) was used to perform CAM assay as previously described.^53^ A approximately 1.0 cm in diameter window was opened in the eggshell to expose the CAM. A 0.5 cm in diameter sterile rubber ring was placed on the CAM before conditioned medium (CM; 100 μL) was added. A piece of sterile adhesive tape was used to close the window, and eggs were positioned in a 37°C incubator, the relative humidity of which was 80%–90% for 2–3 days. Cell culture supernatants of different experimental groups were applied inside the ring at an interval of 24 h. CAMs were fixed with a stationary solution (acetone:methanol = 1:1) for 15 min before being cut and harvested. We counted the number of second‐ and third‐order vessels to assess the effects of CM on angiogenesis.

### 5.14 HUVEC tube formation assay

The tubule formation assay of HUVECs was performed as described previously.^54^ Briefly, Matrigel (200 μL; BD Biosciences) was pipetted into each well of 24‐well plates and polymerized at 37°C for 30 min. HUVECs (2 × 10^4^) were incubated in 200 μL of CM at 37°C in 5% CO_2_ for 6 h before imaging. Pictures were taken under a 100× bright‐field microscope, and the number of the completed tubule structure was used to quantify capillary tubes. Three independent experiments were done for each treatment.

### 5.15 Statistical analysis

All statistical analyses of the experimental data were conducted using SPSS Statistics version 20.0 (IBM Corp.). Differences between groups were identified using a two‐tailed Student's *t* test. The survival curves of CRC patients in the high‐ and low‐*SEMA3B‐AS1* groups were analyzed with the Kaplan–Meier method, and the log‐rank test was used to compare differences. The Pearson correlation coefficient was used to express correlation analyses. *p < 0.05, **p < 0.01, and ***p < 0.001 were considered statistically significant.

## AUTHOR CONTRIBUTIONS

Yi‐Qing Wang, Hui Chen, Cong‐Rui Liao, Yue Han, Anran Xu, Shuang Xu, Qing‐Yuan Li, and Ling‐Ying Zhan contributed to the experimental procedures. Yi‐Qing Wang and Shuang Wang designed the research and analyzed the data. Min‐Hui Yang prepared the figures. Sha‐Sha Hu, Li Zhao, and Lan Wang contributed to the statistical analysis. Yi‐Qing Wang, Yan‐Qing Ding, and Shuang Wang supervised all the work. All authors read and approved the final manuscript.

## CONFLICT OF INTEREST STATEMENT

The authors declare that no conflicts of interest exist.

## ETHICS STATEMENT

The collection of human specimens was approved by the Ethics Committee of Nanfang Hospital, Southern Medical University (Guangdong, China) (NFEC‐2021‐108). Written informed consent was obtained from all participants. All animal experiments were approved by the Committee on the Ethics of Animal Experiments of Southern Medical University (SCXK‐2021‐0041). The protocol was carried out in strict accordance with the recommendations in the Guide for the Care and Use of Laboratory Animals of the National Institutes of Health.

## Supporting information

Supporting Information

Supporting Information

Supporting Information

Supporting Information

## Data Availability

The data that support the findings of this study are available from the corresponding author upon reasonable request.
