# Supplementary material for: SEMA3B‐AS1 suppresses colorectal carcinoma progression by inhibiting Semaphorin 3B‐dependent VEGF signaling pathway activation
Source: MedComm (2020). 2023 Sep 10;4(5):e365. doi: 10.1002/mco2.365 (PMC10492924; doi:10.1002/mco2.365)
Supplement: Supplementary file 1 — Supporting Information [file MCO2-4-e365-s003.docx]

***SEMA3B-AS1* suppresses** **colorectal** **carcinoma progression by inhibiting SEMA3B-dependent VEGF** **signalling pathway activation**

Yi-Qing Wang^1,2#^, Hui Chen^1,2#^, Shuang Xu^1,2#^, Cong-Rui Liao^3^, Anran Xu^2^, Yue Han^2^, Min-Hui Yang^1,2^, Li Zhao^2^, Sha-Sha Hu^2^, Lan Wang^2^, Qing-Yuan Li^4^, Ling-Ying Zhan^2^, Yan-Qing Ding^1,2*^ and Shuang Wang^1,2*^

1. Department of Pathology, Nanfang Hospital, Southern Medical University, Guangzhou, Guangdong, China
2. Department of Pathology, School of Basic Medical Sciences, Southern Medical University, Guangzhou, Guangdong, China

3 Division of Spine Surgery, Department of Orthopaedics, Nanfang Hospital, Southern Medical University, Guangzhou, Guangdong, China

4 Guangdong Provincial Key Laboratory of Gastroenterology, Department of Gastroenterology, Nanfang Hospital, Southern Medical University, Guangzhou, Guangdong, China

#These authors contributed equally to this work.

*Correspondence to:

Shuang Wang, Department of Pathology, Nanfang Hospital, Southern Medical University, Guangzhou 510515, China; Email: shuangw@126.com; Yan-Qing Ding, Department of Pathology, Nanfang Hospital, Southern Medical University, Guangzhou 510515, China; Email: dyqgz@126.com.

**Supplementary Materials and Methods**

#### **ISH and** **evaluation of *SEMA3B-AS1* staining**

The 3-μm-thick paraffin-embedded sections were deparaffinized with xylene for 30 minutes and rehydrated with dilute ethanol of reagent grade from100% to 70%. The tissues were incubated with proteinase K at 37 °C for 20 minutes. Subsequently, the hybridization solution which contained 125 nM of a locked nucleic acid-modified, 5’- digoxigenin (DIG)-labelled oligonucleotide probe of *SEMA3B-AS1* was used to hybridize at a temperature of 51 °C for 12 hours. Then the sections were incubated in an alkaline phosphate conjugated anti-DIG antibody (Roche, Mannheim, Germany) at 37 °C for 1 hours, washed in staining solution, and incubated in NBT/BCIP developing solution (Roche) at 37 °C for 2 hours. A relatively simple and reproducible scoring method10 was used to assess the staining for *SEMA3B-AS1*. The ISH-stained tissue sections were blindly reviewed and scored by two pathologists separately. Additional details are described in the Supplementary Information.

**Evaluation of ISH staining of *SEMA3B-AS1***

To assess the patients’ clinical characteristics, the ISH stained tissue sections were reviewed and scored separately by two blinded pathologists. Staining for *SEMA3B-AS1* was assessed using a relatively simple, reproducible scoring method. On a scale of 0 to 3, the staining intensity was scored as follows: negative (no staining, 0), weak (light blue, 1), medium (blue, 2), or strong (dark blue, 3). The extent of the staining is defined as the percentage of positive staining areas of tumor cells or normal colonic epithelial cells in relation to the whole tumor area or entire section for the normal samples. The extent of staining was scored on a scale of 0 to 4 as follows: 0, 0%; 1, 1–25%; 2, 26–50%; 3, 51–75%; and 4, 76–100%. The sum of the staining-intensity and staining-extent scores was used as the final staining score for *SEMA3B-AS1* (0–7). For statistical analysis, a final staining score of < 3 was considered to denote low-expression of *SEMA3B-AS1* (n=76), and a final staining score of ≥ 3 was considered to denote high-expression of *SEMA3B-AS1* (n=70)*.*

**Cell proliferation assay and colony formation assay**

Cells were seeded in 96-well plates at 0.8~1 × 10^3^ per well. Cell proliferation was evaluated using Cell Counting Kit-8 (CCK-8, Dojindo, Rockville, USA) according to the manufacturer's instructions. For performing colony formation assay, the cells were plated in 6-well plates at 800 per well and maintained in RPMI1640 containing 10% FBS for 2 weeks. After 2 weeks, the cells were washed twice with PBS, fixed with methanol and stained with 0.5% crystal violet. The number of colonies was counted under a microscope.

**Flow cytometry cell cycle**

Cells were plated in 6-well plates at 5×10^5^ per well. The cell-cycle distribution was analyzed by propidium iodide (Sigma-Aldrich) staining and flow cytometry. All experiments were performed in triplicates.

**Wound healing assays**

Cells were seeded on six-well culture plates at a density of approximately 1 × 10^6^ cells per well and incubated for 24 h (80–90% confluence). A 10-μL plastic pipette tip was used to scratch the monolayers. The wounded cells were then cultured in a serum-free medium for 48 h.Three different microscopic fields (× 200) were selected randomly. Cell motility was quantified by measuring the distance (μm) between the edge of the scratch wound was measured before and after cell migration. Experiments were carried out in triplicate and repeated at least 3 times.

**Cell invasion analysis**

For the cell invasion assay, matrigel-coated chambers (BD Biosciences, San José, CA, USA) containing 8 μm pores were used. Tumor cells or Human umbilical vein endothelial cells were seeded into the upper chambers (coated in matrigel) by maintaining a concentration of 2 × 10^5^ in serum-free medium. The lower chamber of the transwell was filled with culture media containing 10% FBS as a chemo-attractant. Then, the chambers were incubated at 37°C for 48 hours. For quantification, the cells were fixed with 4% paraformaldehyde for 20 min, stained with hematoxylin for 20 min at room temperature, and counted in five randomly chosen fields (× 200) under a microscope.

#### Tumorigenic and metastasis assays *in vivo*

For in vivo tumorigenicity, a total of 5~6×10^6^ HCT116 cells with *SEMA3B-AS1* or SEMA3B overexpression and control cells were trypsinized, counted, resuspended in sterile PBS, and then injected subcutaneously into the right and left bilateral upper limbs of mice. The tumour volume and overall health of the mice were monitored for 21 days. Tumour volume was calculated according to the formula 0.5 × length × width2. The length and width of the tumour were measured by a Vernier calliper. Each experimental group contained seven mice.

For the metastatic mouse model assay, the nude mice of the experimental group were anaesthetized with 1% pentobarbital, and 5 × 10^6^ cells were injected under the splenic capsule. The general growth conditions of the mice were observed after surgery to determine whether there were signs of cachexia, such as wasting or arched backs. The mice were sacrificed within 28 days after the operation, their individual spleens and livers were excised for photographing, and metastases were observed by histological analysis. Tissues were then fixed with formaldehyde and paraffin-embedded, and 3-mm sections were cut and stained with haematoxylin and eosin (H&E).

#### Western blotting analysis

Cells and tissues were lysed on ice for 15 minutes in prechilled RIPA buffer (1 M Tris-HCl [pH 7.5], 150 mM NaCl, 0.1%SDS, 0.1% Triton X-100 ,0.25% sodium deoxycholate, and 0.1% NP-40) supplemented with protease inhibitors (Roche, Basel, Switzerland). Protein lysates were boiled for 10 minutes, quantified and loaded onto 8~15% SDS–PAGE gels for separation and then transferred to polyvinylidene fluoride (PVDF) membranes (Millipore, Darmstadt, Germany). The membranes were blocked with 5% skimmed milk in Tris-buffered saline containing 0.1% Tween 20 (TBST) and incubated at 4 °C for 8 hours with the following primary antibodies: anti‐SEMA3B (1:1000, NB100-2218SS), anti‐NRP1 (1:1000, ab81321, Abcam, Cambridge, UK), anti‐EP300 (1:1000, ab14984), anti‐H3K9ac (1:500, ab32129), anti‐VEGF (1:1000, AF5131, Affinity, Dallas, Texas, UK), anti‐Caspase 3 (1:1000, 9662S, Cell Signaling Technology, Boston, USA), anti‐Cleaved caspase3 (1:1000, 9664S, Cell Signaling Technology), anti‐Caspase 9 (1:1000, 9504S, Cell Signaling Technology) or anti‐Cleaved caspase9 (1:1000, 9509S, Cell Signaling Technology). Anti‐histone 3 (1:1000, 17168-1-AP, Proteintech Group), anti‐α-tubulin (1:1000, 11224-1-AP, Proteintech Group) and anti‐GAPDH (1:1000, 60004-1-lg, Proteintech Group) were used as protein‐loading controls. After 3 washes with TBST, the membranes were incubated for one hour at 37 °C with HRP-conjugated secondary antibodies and washed again, and the signal was visualized with ECL western blotting substrate (Thermo Fisher Scientific) as described by the manufacturer. Quantity One Software (Bio‐Rad, West Berkeley, CA, USA) was used to detect the immunoblot signals by densitometry.

#### RNA pull-down assays

#### Biotin-labelled SEMA3B-AS1 and its antisense RNA were in vitro transcribed from vector pLenti-EF1a-EGFP-F2A-Puro-CMV-MCS-SEMA3B-AS1 with Biotin RNA Labelling Mix (Roche Diagnostics, Indianapolis, IN) and in vitro transcribed using T7 RNA polymerase (MEGAscript Kits, Ambion, USA). Biotinylated RNA was heated to 90 °C for 2 minutes, placed on ice for 2 minutes, supplied with RNA structure buffer (10 mM Tris-HCl [pH 7.0], 0.1 M KCl and 10 mM MgCl2) and then moved to room temperature for 20 minutes to allow for proper secondary structure formation. The protein extracted from HCT116 cells was mixed with biotinylated RNAs. Then, the protein–RNA mixture was incubated with streptavidin magnetic beads (Life Technologies, Carlsbad, CA, USA) and washed. The retrieved proteins were boiled for 10 minutes at 100 °C and then analysed by western blotting assay. RNA input was detected using streptavidin-HRP by dot-blot assay.

#### RNA immunoprecipitation (RIP)

#### RIP assays were conducted according to the instructions provided by the manufacturer (Merck Millipore 17-700 Magna RIP Kit, Bedford, USA). Briefly, cells were cross-linked with 1% (w/v) formaldehyde, quenched with 1/10 volume of 1.25 M glycine in PBS, washed with PBS, harvested by scraping, centrifuged and then resuspended in lysis buffer containing an RNase inhibitor and protease inhibitor cocktail. Protein A magnetic beads were preincubated with anti-rabbit EP300 or anti-rabbit IgG for 30 minutes at room temperature, and lysates were then immunoprecipitated with the beads overnight at 4 °C. Crosslinks were reversed at 65 °C for 3 hours, and proteins were digested with 1 mg/ml proteinase K at 55 °C for 2 hours. RNA was purified using TRIzol from RNA–protein complexes that bound to the beads and then analysed by qRT-PCR.

#### Chromatin immunoprecipitation (ChIP) assay

ChIP assays were performed with a kit (#17–10086, Merck), and all experimental procedures were performed according to manufacturer instructions. Briefly, cells (1 × 107) in a 10 cm culture dish were treated with 1% formaldehyde to cross-link chromatin-associated proteins to DNA. The cell lysates were subjected to ultrasound for 9–10 sets of 10 s pulses at 40% output to shear the DNA into fragments between 200 and 1000 bps. Equal cell lysates were incubated with EP300 antibody (Abcam, ab275378) or normal rabbit IgG antibody as a negative control. Both the above chromatin supernatant-antibody mixtures were incubated with 20 μL magnetic protein A/G beads overnight at 4 °C with rotation. The human SEMA3B promoter was amplified by PCR and qRT–PCR. All ChIP assays were performed in triplicate.

#### Immunofluorescence

Paraffin-embedded specimens were cut into 3-μm sections and baked at 65 °C for 30 min. The sections were deparaffinized with xylene and rehydrated. After treatment with 3% hydrogen peroxide in methanol to quench endogenous peroxidase activity, the sections were submerged in citrate buffer and high-pressure boiled for antigen retrieval, followed by incubation with 1% bovine serum albumin to block nonspecific binding. Rabbit anti-SEMA3B (1:200, NB100-2218SS) or rabbit anti-NRP1 (1:200, ab81321) was incubated with the sections overnight at 4 °C. The next day, slides were incubated with Alexa Fluor 488- and Alexa Fluor 594-labelled secondary antibodies (1:1000, Proteintech Group Inc, Wuhan, China) for 1 h at room temperature. To visualize nuclei, slides were incubated with 6-diamidino-2-phenylindole (DAPI; KeyGEN, Nanjing, China). Samples were mounted with ProLong Gold antifade reagent (Life Technologies) and imaged on a confocal microscope (× 400).

**Supplementary figures**

**
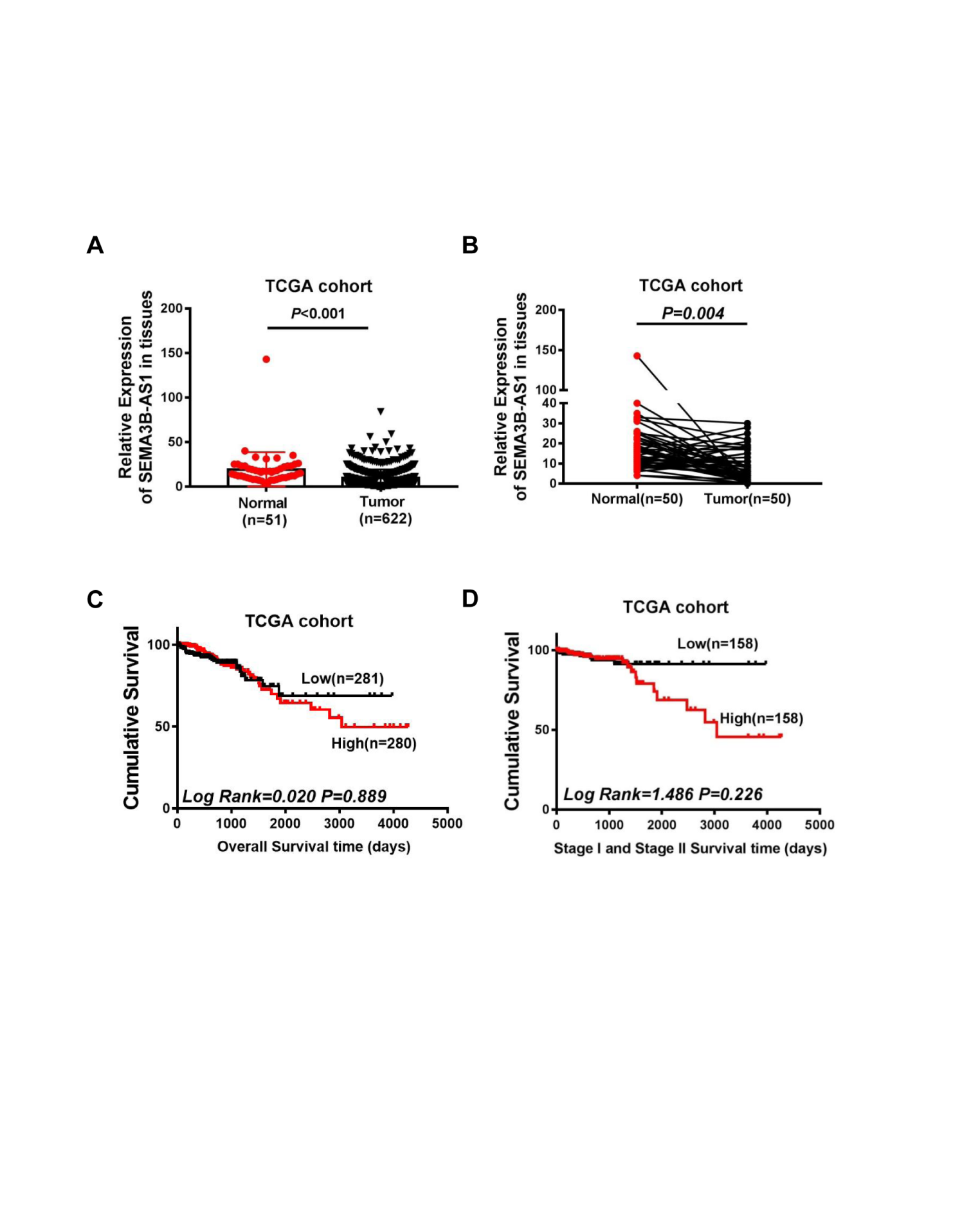
**

**Figure S1: *SEMA3B-A*S1 is downregulated and could be an important prognostic factor for the TNM stage of patients with colorectal carcinoma according to TCGA cohort.** (A-B), The level of *SEMA3B-AS1* in unpaired (A) and paired (B) colorectal carcinoma and noncancerous tissue samples from the TCGA cohort. (C-D), Kaplan–Meier survival analysis in all patients (C) and early-stage patients (D) with colorectal carcinoma according to *SEMA3B-AS1* expression in the TCGA cohort.


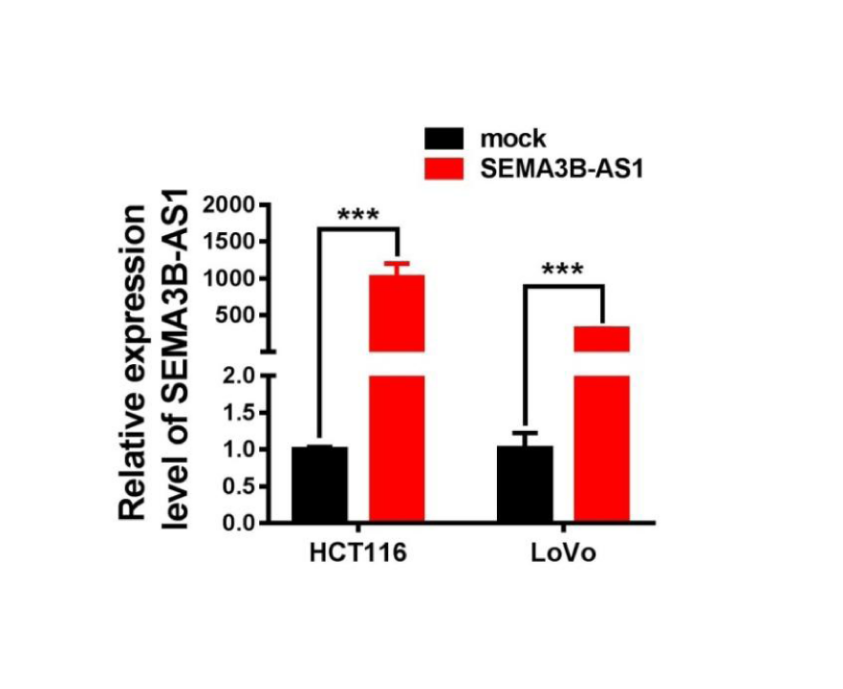


**Figure S2: HCT116 and LoVo cells were infected with plasmid to establish two cell lines with stable overexpression of *SEMA3B-AS1*.**

**
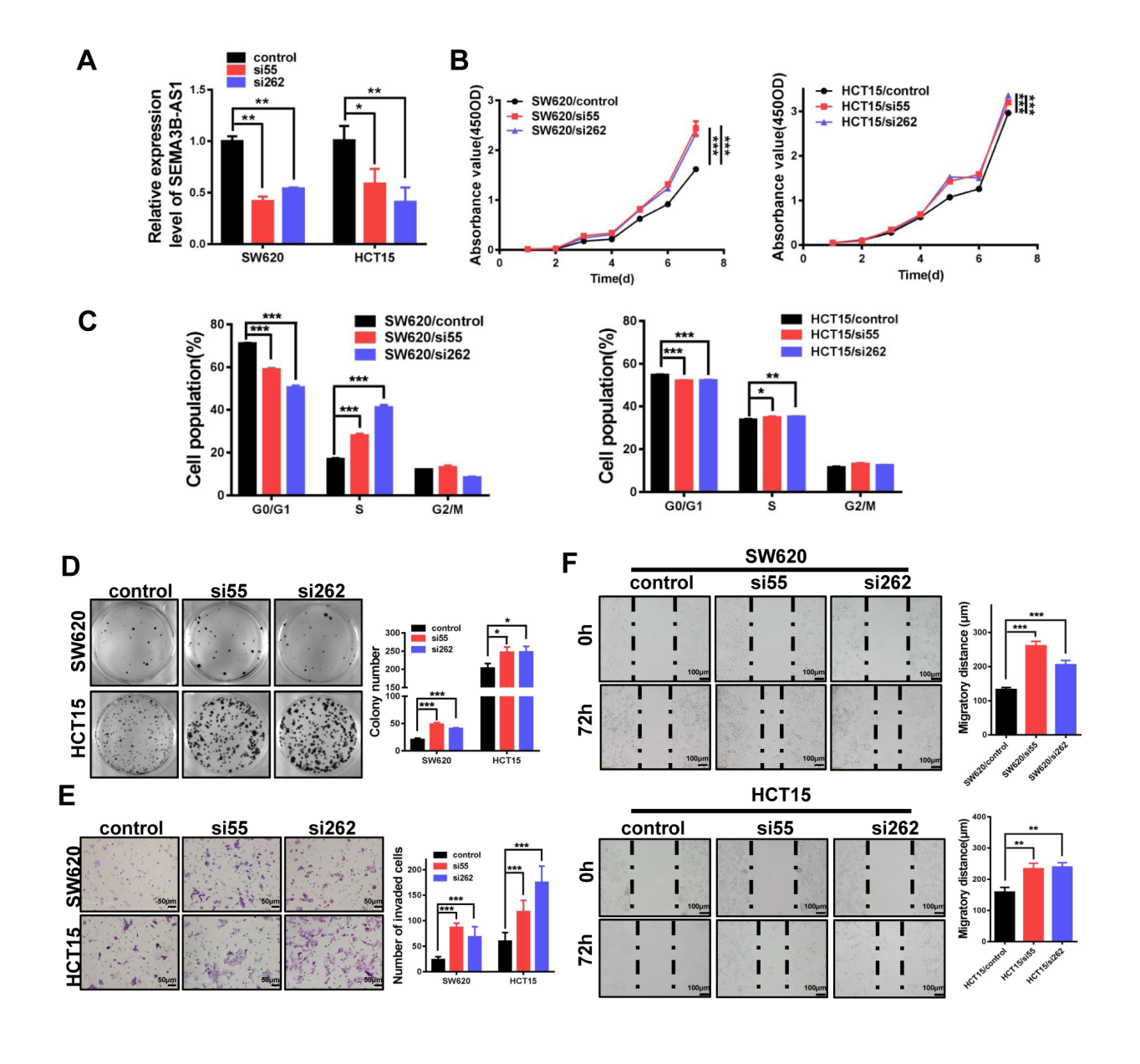
**

**Figure S3: *SEMA3B-A*S1 downregulation promotes colorectal carcinoma cell growth and metastasis *in vitro*.** A, *SEMA3B-AS*1 levels in SW620 and HCT15 cells after siRNA-mediated knockdown of *SEMA3B-AS1* were detected by real-time RT–PCR. B, *SEMA3B-AS1* downregulation promoted cell proliferation in colorectal carcinoma cell lines as determined by CCK-8 assay. C, *SEMA3B-AS1* downregulation promoted cell cycle arrest in the G1 phase in colorectal carcinoma cells. D, *SEMA3B-AS1* downregulation promoted colony formation in colorectal carcinoma cells. Representative images (left) and quantitative analyses (right) are shown. E, *SEMA3B-AS1* downregulation promoted colorectal carcinoma cell invasion in a Matrigel invasion assay. Scale bars indicate 50μm. F, *SEMA3B-AS1* downregulation promoted cell migration in the wound-healing assay. Scale bars indicate 100μm.The experiments were performed at least three times, and the data are expressed as the mean ± SD.


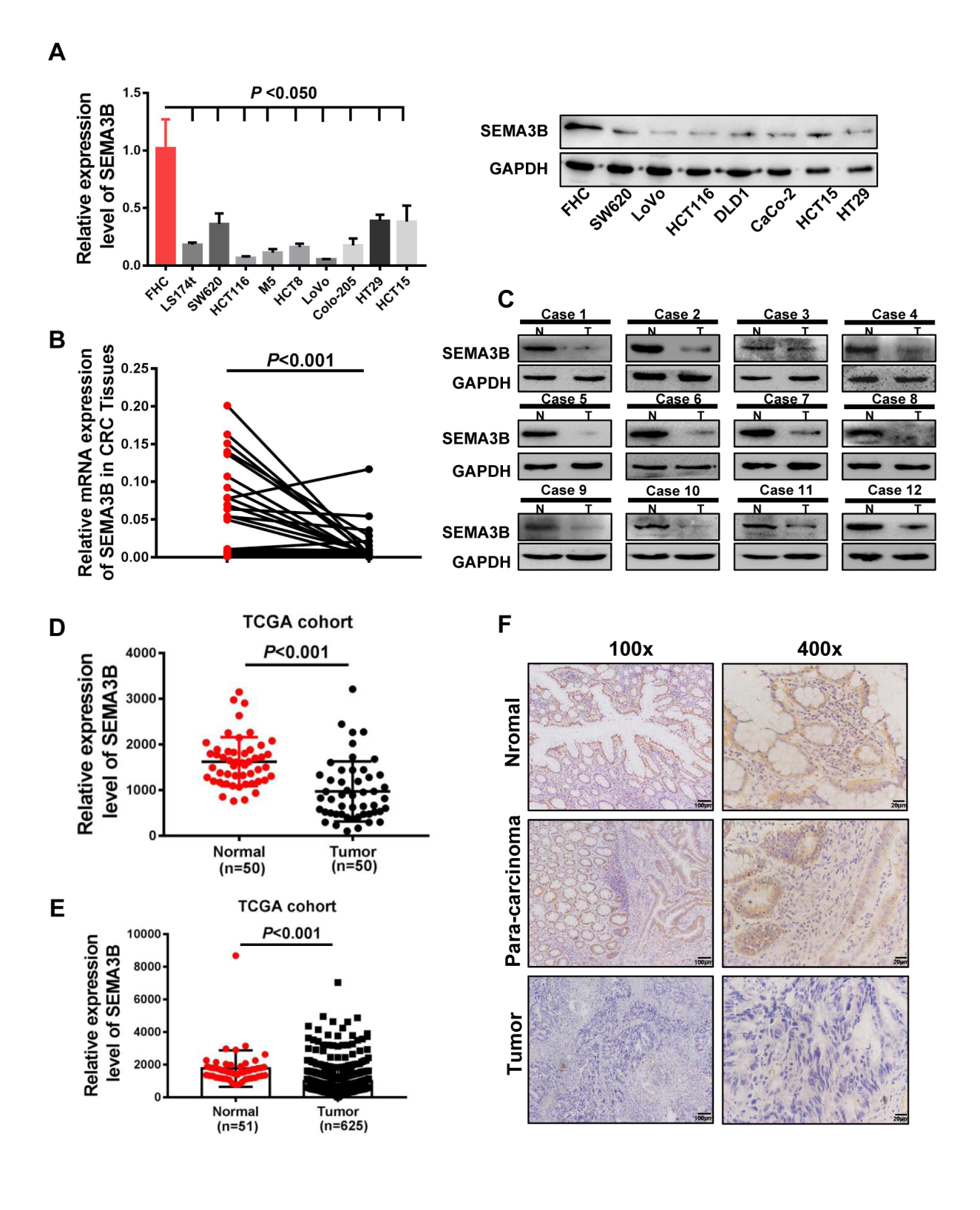


**Figure S4: SEMA3B is downregulated in colorectal carcinoma. A**, The mRNA (left) and protein (right) levels of SEMA3B in colorectal carcinoma and colon mucosa epithelial (FHC) cell lines. B-C, The level of SEMA3B in paired colorectal carcinoma and adjacent noncancerous tissues by real-time PCR (B) and western blotting (C). D-E, The level of SEMA3B in paired (D) and unpaired (E) colorectal carcinoma and noncancerous tissue samples from the TCGA cohort. F, Expression analysis of SEMA3B in normal colorectal mucosa and colorectal carcinoma tissues by IHC. Scale bars indicate 100μm (left column) and 20μm (right column).


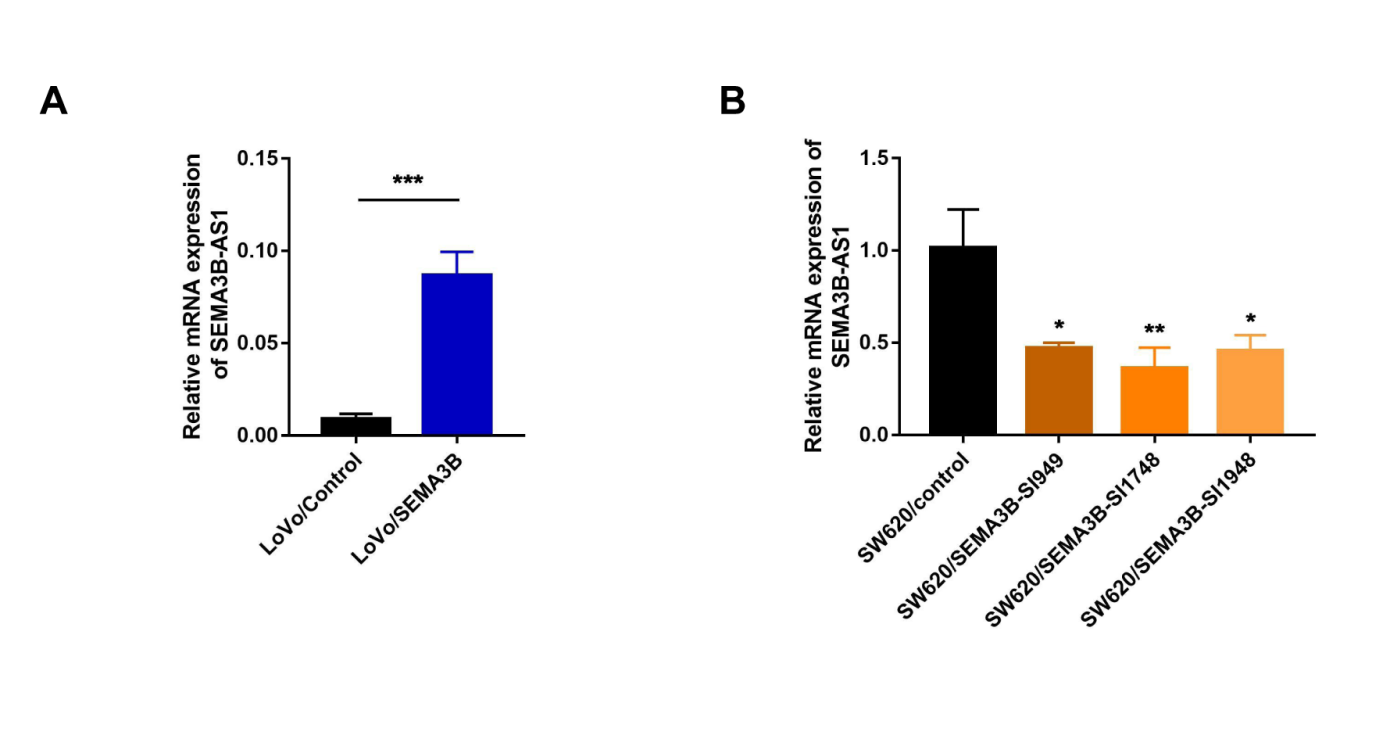


**Figure S5: The expression of *SEMA3B-AS1* changes when SEMA3B was upregulated (A) or downregulated (B).**

**
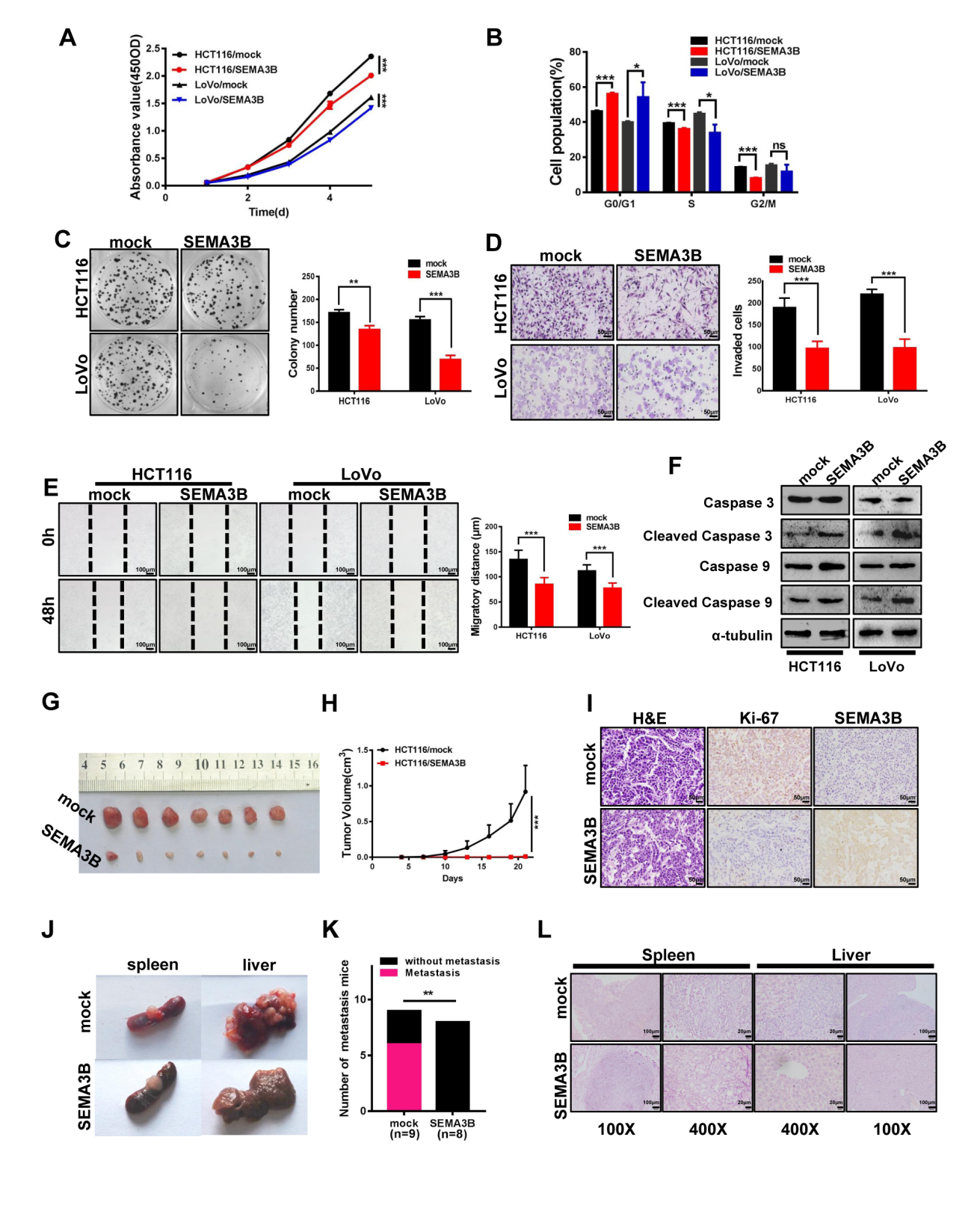
**

**Figure S6: SEMA3B overexpression inhibits colorectal carcinoma cell growth and metastasis *in vitro* and *in vivo*.** A, SEMA3B overexpression suppressed cell proliferation in colorectal carcinoma cell lines as determined by CCK-8 assay. B, SEMA3B overexpression induced cell cycle arrest in the G1 phase in colorectal carcinoma cells. C, SEMA3B overexpression inhibited colony formation in colorectal carcinoma cells. Representative images (left) and quantitative analyses (right) are shown. D, SEMA3B overexpression inhibited colorectal carcinoma cell invasion in a Matrigel invasion assay. Scale bars indicate 50μm. E, SEMA3B overexpression suppressed cell migration in the wound-healing assay. Scale bars indicate 100μm. The experiments were performed at least three times, and the data are expressed as the mean ± SD. F, Apoptosis assays were performed to determine the effects of SEMA3B overexpression in CRC cells by western blot. G-H, SEMA3B overexpression inhibited subcutaneous tumour formation in nude mice. HCT116 cells with ectopic overexpression of SEMA3B and control cells were inoculated into nude mice (n = 7 per group). The tumour xenografts (G) are shown after ectopic-subcutaneous implantation for 3 weeks. The effect of SEMA3B on colorectal carcinoma tumour growth was evaluated based on tumour volume in the two groups (H). I, Representative photographs of haematoxylin and eosin (H&E) and IHC staining for Ki-67 and SEMA3B antibodies in primary cancer tissues. Scale bars indicate 20μm. J-L, Intrasplenic injections to establish a liver metastasis model in nude mice. The tumours in spleen and liver metastases after CRC cell intrasplenic injections for 6 weeks (J) and the statistical distribution of metastasis numbers (K) are shown. The tissues were stained by H&E staining (L). Scale bars indicate 100μm (100X) and 20μm (400X). *, P < 0.05; **, P < 0.01; ***, P < 0.001.


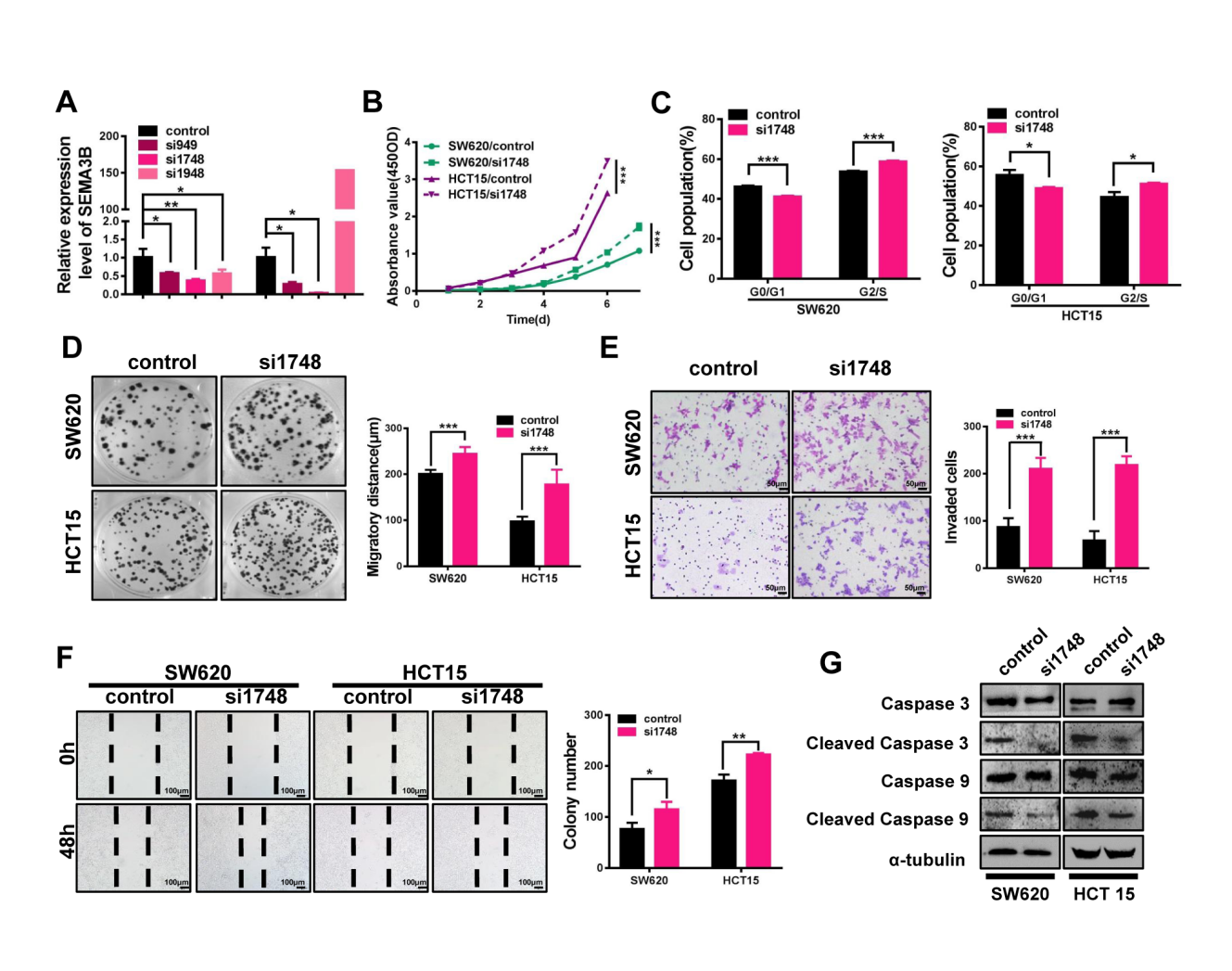


**Figure S7: SEMA3B downregulation promotes colorectal carcinoma cell growth and metastasis *in vitro*.** A, SEMA3B levels in SW620 and HCT15 cells after siRNA-mediated knockdown of SEMA3B were detected by real-time RT–PCR. B, SEMA3B downregulation promoted cell proliferation in colorectal carcinoma cell lines as determined by CCK-8 assay. C, SEMA3B downregulation promoted cell cycle arrest in the G1 phase in colorectal carcinoma cells. D, SEMA3B downregulation promoted colony formation in colorectal carcinoma cells. Representative images (left) and quantitative analyses (right) are shown. E, SEMA3B downregulation promoted colorectal carcinoma cell invasion in the Matrigel invasion assay. Scale bars indicate 50μm. F, SEMA3B downregulation promoted cell migration in the wound-healing assay. Scale bars indicate 100μm. G, Apoptosis assays were performed to determine the effects of SEMA3B knockdown in CRC cells by western blot. The experiments were performed at least three times, and the data are expressed as the mean ± SD. * P<0.05, ** P<0.01, *** P<0.001.


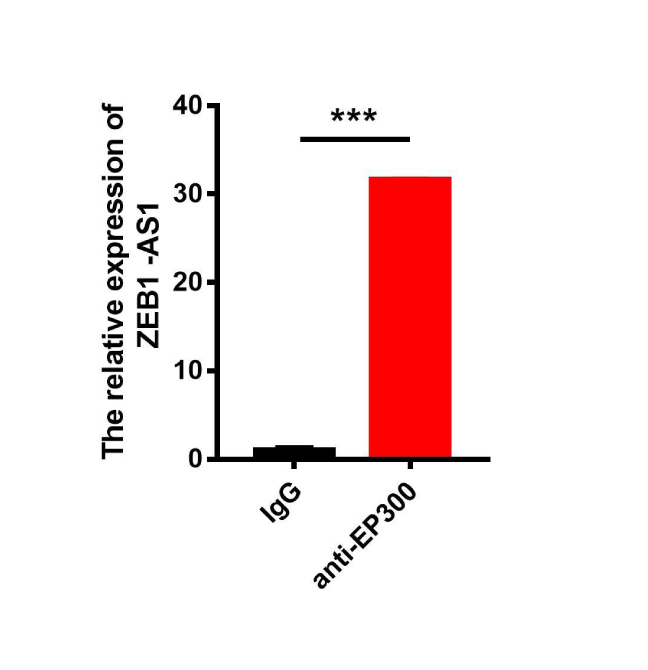


**Figure S8: RIP experiments were performed using an EP300 or nonspecific IgG antibody to determine the amount of ZEB1-AS1.**

**
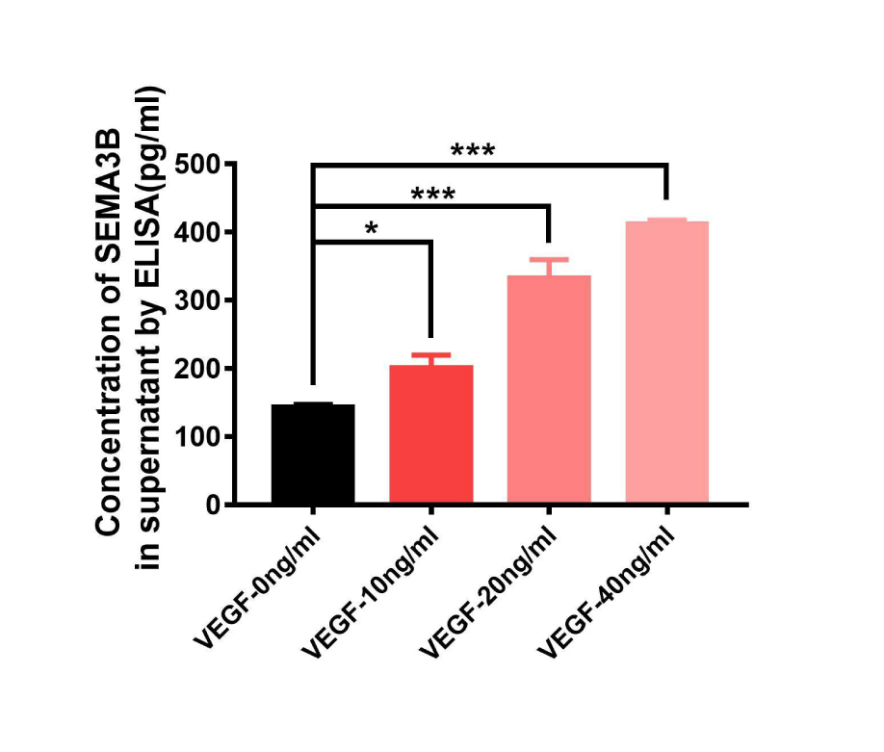
**

**Figure S9:** **The content of SEMA3B increased in the cell culture supernatant with increasing exogenous VEGF concentration.**
